# Voluntary wheel running exercise attenuates VPA-induced ASD-like behaviors in male rats: implication of the vagal pathway of the gut-brain axis

**DOI:** 10.1038/s41522-026-00962-4

**Published:** 2026-03-20

**Authors:** Yinhua Li, Jiugen Zhong, Yingying Shen, Jiaheng Gong, Yanqing Feng, Wanting Lan, Xiaohui Hou

**Affiliations:** 1 Sport and Health Department, Guangzhou Sport University, Guangzhou, China; 2 College of Rehabilitation, Shenyang Medical College, Shenyang, China; 3 School of Exercise and Health, Shanghai University of Sport, Shanghai, China; 4 Medical School of Shaoxing University, Shaoxing, China; 5 Guangdong Key Laboratory of Human Sports Performance Science, Guangzhou Sport University, Guangzhou, China; 6 Guangdong Provincial Key Laboratory of Physical Activity and Health Promotion, Guangzhou Sport University, Guangzhou, China

**Keywords:** Diseases, Microbiology, Neurology, Neuroscience

## Abstract

Autism spectrum disorder (ASD) is a prevalent neurodevelopmental disorder with elusive pathogenesis and lack of targeted therapies. While exercise can ameliorate ASD-like behaviors, its underlying mechanisms remain unclear. Recent studies have identified dysbiosis of gut microbiota and altered levels of short-chain fatty acids (SCFAs), as critical contributors to ASD-associated behavioral abnormalities. This study investigated the potential role of the gut-brain axis, specifically the vagal pathway, in mediating the therapeutic effects of voluntary wheel running exercise in a valproic acid (VPA)-induced ASD-like rat models. We demonstrated that six weeks of voluntary wheel running exercise attenuated ASD-like behavioral deficits. Exercise restructured gut microbial communities and elevated SCFA levels, notably butyrate, in feces and plasma. Concurrently, exercise normalized imbalances of neuroactive substances in the hippocampus and prefrontal cortex and suppressed neuroinflammation, evidenced by reduced microglial/astrocytic reactivity and a shift in microglial polarization toward an anti-inflammatory phenotype. Critically, subdiaphragmatic vagotomy attenuated these exercise-induced improvements, including the restoration of neuroactive substance homeostasis, resolution of neuroinflammation, and the amelioration of behavioral deficits. Our findings suggest that intact vagal signaling plays a critical role in coordinating gut-derived microbial and metabolic signals with central neuroadaptations to mediate the benefits of voluntary exercise on ASD-like behaviors.

## Introduction

Autism spectrum disorder (ASD) is a heterogeneous neurodevelopmental condition characterized by core symptoms of impaired social communication, restricted interests, and repetitive/stereotyped behaviors^1,2^. The worldwide prevalence is estimated to be 1–2%, meaning that at least 78 million people in the world have ASD^3^. Typically emerging in early childhood, ASD can lead to lifelong social dysfunction^4^. The multifactorial etiology of ASD remains elusive, involving complex interactions between genetic susceptibility and environmental determinants^5^.

Recent evidence highlights that approximately 40% of children with ASD exhibit comorbid gastrointestinal (GI) dysfunction^6–8^, with GI symptom severity correlating closely with ASD behavioral manifestations^9^. Both clinical investigations^10,11^ and preclinical models^12–14^ consistently demonstrate ASD-associated gut microbiota dysbiosis, marked by altered microbial diversity and functional profiles^10,15^. Fecal microbiota transplantation (FMT) in ASD cohorts significantly ameliorated GI distress and core behavioral deficits^16^, with sustained benefits persisting through 2-year follow-up^17^. Microbiota transplantation from ASD donors to germ-free mice recapitulates core behavioral phenotypes, including elevated repetitive behaviors, impaired social interaction, and reduced locomotor activity^18^. These findings collectively provide compelling evidence for the gut microbiome’s causative role in ASD pathogenesis. In addition, significant alterations in short-chain fatty acids (SCFAs)—key microbial metabolites—have been observed in ASD populations. Studies reveal decreased fecal propionate and butyrate levels in children with ASD^19^, while Liu et al. demonstrated reduced acetate and butyrate concomitant with elevated valerate in constipated ASD subgroups^20^. Conflicting reports exist, with a study showing elevated fecal acetate, butyrate, isobutyrate, and isovalerate in children with ASD^21^. Although current evidence on SCFAs dysregulation remains contradictory, compelling data suggest that microbial-derived SCFAs imbalances may critically contribute to the pathophysiology of ASD, with the underlying mechanisms warranting further investigation.

Accumulating evidence substantiates that gut microbiota and SCFAs communicate bidirectionally with the central nervous system (CNS) via the microbiota-gut-brain axis^22–24^. As the most direct gut-brain pathway, the vagus nerve plays a significant role in mediating microbial influences on emotional and behavioral responses^25,26^. A study by Sgritta et al. demonstrated that bilateral subdiaphragmatic vagotomy eliminates the beneficial effect of *Lactobacillus reuteri* on social deficits in ASD model mice^12^. Gut-derived SCFAs have been shown to modulate CNS function by activating free fatty acid receptor (FFAR) on vagal afferent^24^. Vagus nerve stimulation (VNS) has demonstrated therapeutic efficacy in ameliorating ASD-associated behavioral deficits^27–29^. VNS can suppress pro-inflammatory cytokine production by microglia and modulate their activation states^30–32^. Concurrently, VNS activates the cholinergic anti-inflammatory pathway, thereby alleviating neuroinflammation^33–35^. VNS can also restore neurotransmitter homeostasis in the brain, exerting neuroprotective effects that regulate emotional and cognitive functions^36^.

Physical exercise has attracted attention as a potential therapeutic strategy for abnormal behaviors in ASD^37–40^. Our preliminary observations suggest that aerobic training may restore microbial diversity in children with ASD and shift gut microbiota structure toward neurotypical patterns, with parallel improvements in core symptoms. Furthermore, our preclinical investigations indicated that voluntary wheel running exercise modifies gut microbial composition and fecal SCFAs profiles in ASD model rats, correlating with behavioral amelioration. However, the molecular mechanisms by which exercise improves ASD symptoms require further investigation.

Therefore, in the present study, we performed subdiaphragmatic vagotomy on valproic acid (VPA)-induced ASD model rats and subjected them to voluntary wheel running exercise. We focused our central analysis on the prefrontal cortex (PFC) and the hippocampus, given their well-established roles in regulating social behavior, cognition, and emotion—core domains affected in ASD—and their known sensitivity to both neuroinflammatory and gut-brain axis signaling^41–43^. Using multidisciplinary approaches including 16S rRNA amplicon sequencing, targeted metabolomics, immunohistochemical analyses, and behavioral assessments, we aim to elucidate the pivotal role of the vagal pathway of the gut-brain axis in exercise-induced regulation of central neuroactive substance levels, reduction of neuroinflammation, and improvement of ASD-like behaviors.

## Results

### Voluntary wheel running exercise attenuated VPA-induced ASD-like behaviors in rats

**Fig. 1:**
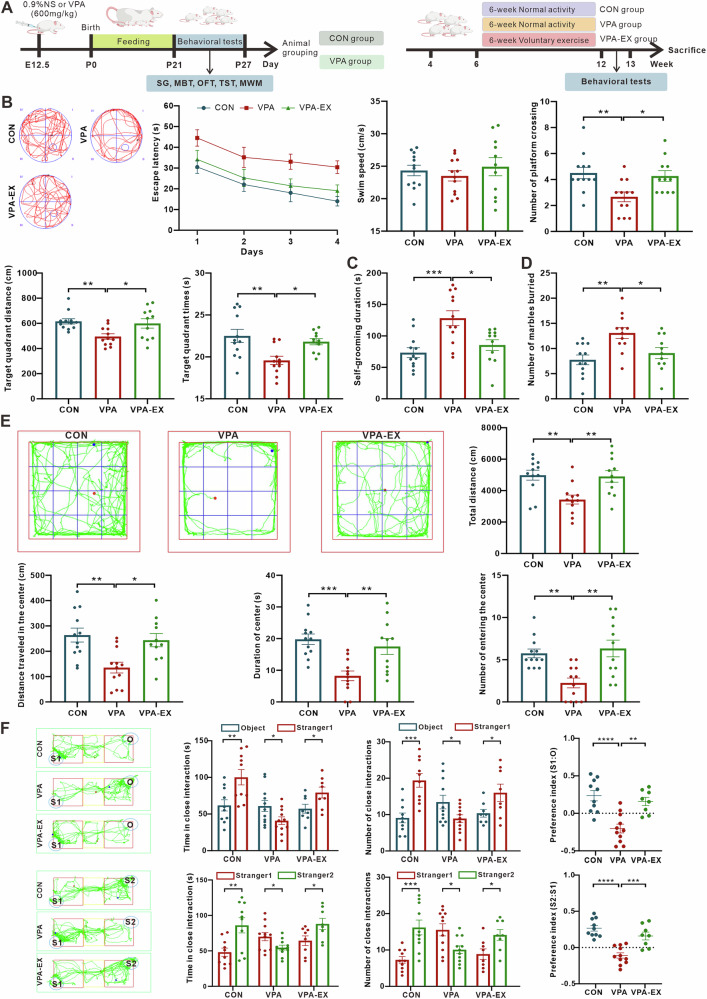
Effects of voluntary running wheel exercise on ASD-like behaviors. **A** Timeline of the experimental procedure. **B** The escape latency in the learning phase, the swim speed, the number of exact crossings over the previously hidden platform in the probe phase, and the distance and time in the target quadrant in the probe phase in the Morris water maze test (MWM). **C** Duration in self-grooming behavior (SG). **D** The number of buried marbles in the marble burying test (MBT). **E** The total distance traveled, distance traveled in the center, time spent in the center, and the number of entering the center in the open-field test (OFT). **F** The time and number of times spent with Object (O), Stranger 1 (S1) and Stranger 2 (S2), and the preference indexes in the three-chamber social test (TST). ^*^P < 0.05, ^**^P < 0.01^, ***^P < 0.001, ^****^P < 0.0001.

Extensive evidence demonstrates that prenatal VPA exposure in rodents induces behavioral abnormalities, including impaired social interaction, increased repetitive behaviors, and elevated anxiety levels—core features recapitulating clinical manifestations observed in human ASD patients^9,44^. Given the well-established validity of maternal VPA exposure for modeling ASD-like phenotypes in rodents^45^, we established an ASD animal model by injecting VPA into pregnant rats at embryonic day 12.5 (E12.5). To evaluate the therapeutic potential of exercise, VPA-induced ASD-like rats underwent a 6-week voluntary wheel running exercise intervention (Fig. 1A).

Morris water maze tests (Fig. 1B) revealed significant cognitive deficits in VPA-induced ASD-like rats compared to the CONN group, as evidenced by markedly prolonged escape latency, accompanied by reduced number of platform crossings, decreased distance in the target quadrant, and diminished time spent in the target quadrant. 6-week voluntary running wheel exercise ameliorated these deficits, demonstrating shortened escape latency, along with increased number of platform crossings, extended target quadrant distance, and prolonged target quadrant duration, suggesting exercise enhanced learning memory and spatial navigation.

To investigate the effects of exercise on repetitive and stereotypic behaviors characteristic of VPA-induced ASD-like rats, we employed self-grooming analysis and marble burying tests. Self-grooming analysis (Fig. 1C) showed that VPA rats exhibited significantly prolonged self-grooming duration compared to the CONN group, indicative of heightened repetitive behavior. The marble burying test (Fig. 1D) further confirmed hyperactive stereotypic behavior in VPA rats, which buried significantly more marbles than their CONN counterparts. Six weeks of voluntary wheel running exercise substantially attenuated this phenotype, with a marked reduction in grooming duration and the number of buried marbles.

Open field test (Fig. 1E) revealed that compared with CONN rats, VPA rats exhibited significantly reduced spontaneous locomotion, as evidenced by decreased total distance traveled and reduced average speed (Supplementary Fig. 1). Concomitant impaired exploratory behavior occurred, with VPA rats showing marked reductions in central zone exploration metrics: distance traveled in the central zone, duration in the central zone, and number of entering the central zone. Six weeks of voluntary wheel running exercise had the potential effective to improve spontaneous activity and increase exploratory behaviors towards the novel environment in VPA rats.

To assess the effect of exercise on socialization in VPA rats, we performed a three-chamber social test (Fig. 1F). In the sociability test, CONN rats spent significantly more time interacting with the Stranger 1 (S1) than with the metal cage, as expected, reflecting the natural preference of rats to socialize. In contrast, VPA rats exhibited more interaction time with the metal cage than Stranger 1 (S1), indicating impaired sociability. In the social novelty test, CONN rats showed higher preference to the novel Stranger 2 (S1) than the familiar Stranger 1 (S1), but the VPA rats exhibited defective recognition of novel Stranger 2 (S2), demonstrated by reduced sniffing duration, frequency, and preference index. Remarkably, voluntary exercise significantly ameliorated sociability deficits and social novelty recognition impairments.

### Voluntary wheel running exercise restructured the composition of gut microbiota in VPA-induced ASD-like rats

**Fig. 2:**
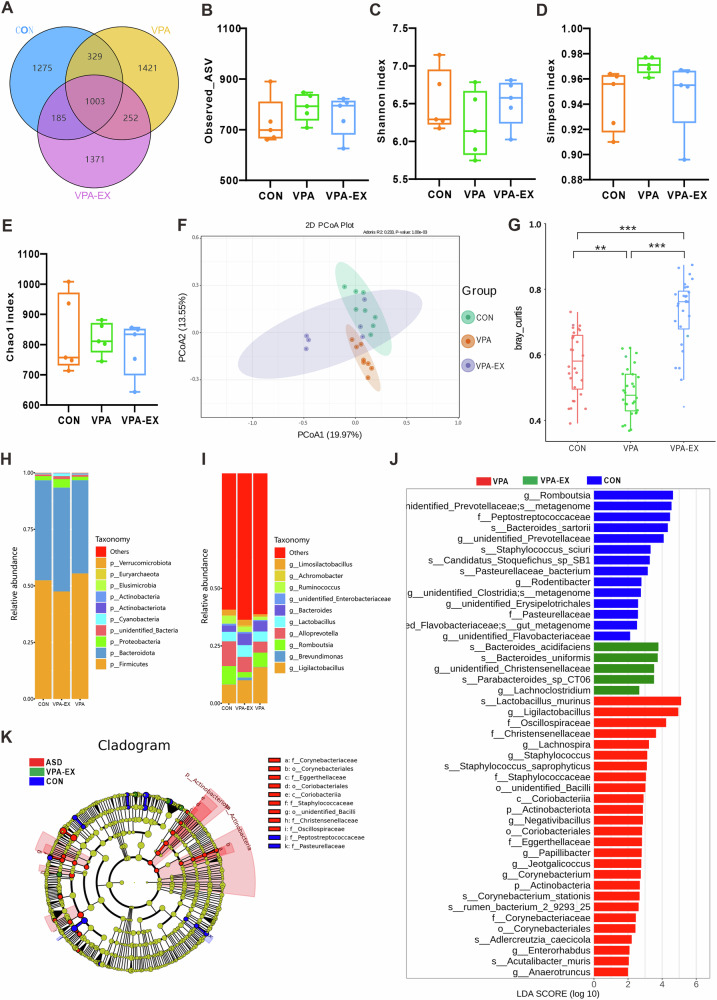
Voluntary wheel running exercise restructured the composition of gut microbiota in VPA-induced ASD-like rats. **A** Venn diagram illustrates the numbers of ASVs in the CONN, VPA, and VPA-EX groups. Alpha diversity is illustrated using a box plot of the **B** Observed_ASV, **C** Shannon, **D** Simpson, and **E** Chao1 indices. **F** Beta-diversity was assessed using PCoA. **(G)** The differences in β diversity among groups were analyzed using Tukey’s test. The top 10 relative abundances of gut microbiota at the phylum (**H**), and genus (**I**) taxonomic levels for the three groups. **J** Histogram of LDA scores for bacterial taxa significantly enriched in gut microbiota from each group (LDA score >2). The LDA score indicates the effect size and ranking of each differentially abundant taxon. **K** Cladogram from the LEfSe illustrating showing differences in fecal taxa. ^*^P < 0.05, ^**^P < 0.01, ^***^P < 0.001, ^****^P < 0.0001.

We performed 16S rRNA gene amplicon sequencing to reveal the effect of exercise on the gut microbiota in VPA-induced ASD-like rats. Venn diagram analysis (Fig. 2A) delineated the shared and unique amplicon sequence variants (ASVs) across CONN, VPA, and VPA-EX groups, highlighting conserved and divergent microbial communities. The CONN, VPA, and VPA-EX groups harbored 1275, 1421, and 1371 unique ASVs, respectively, with 1003 ASVs common to all three groups. Alpha diversity metrics, including Observed_ASV, Chao1, Shannon, and Simpson indices, were employed to assess within-sample microbial community richness and diversity. Chao1 and Observed_ASV quantify species richness, while Shannon and Simpson indices reflect the diversity of species distribution. As illustrated in Fig. 2B–E, the alpha diversity analysis revealed no statistically significant differences in gut microbiota alpha diversity indices across experimental groups. This outcome suggests that 6 weeks of voluntary wheel running exercise exerted no significant impact on the richness or diversity of gut microbial communities in VPA rats. Beta diversity analysis, reflecting inter-sample microbial compositional differences, was performed using principal coordinate analysis (PCoA) based on phylogenetically informed Unifrac distances. Visualization of the primary coordinate axes revealed distinct clustering patterns among groups (Fig. 2F). CONN and VPA groups exhibited significant spatial separation, indicating divergent gut microbiota structures. VPA-EX group clustered separately from VPA counterparts, suggesting exercise-induced alterations of microbial communities. Further beta diversity comparison via Tukey’s post hoc test confirmed significant differences between VPA and both CONN and VPA-EX groups (Fig. 2G), demonstrating that voluntary exercise restores ASD-associated dysbiosis toward a CONN-like microbiota configuration.

To further investigate the effect of exercise on the distribution of species in the gut microbiota of VPA rats, we analyzed the relative abundance of species at the phylum and genus levels in each group. At the phylum level (Fig. 2H), *Firmicutes* and *Bacteroidota* dominated all groups, with *Proteobacteria* showing exercise-associated expansion. At the genus level (Fig. 2I), VPA rats exhibited elevated *Ligilactobacillus*, reduced *Alloprevotella*, and diminished *Romboutsia*. Exercise intervention partially normalized these perturbations, with the VPA-EX group showing moderated *Ligilactobacillus* abundance, recovered *Alloprevotella*, and emergence of beneficial *Lactobacillus*.

The Linear discriminant analysis Effect Size (LEfSe) analysis looks for biomarkers with statistical differences between groups and aggregates species with LDA > 2 into a distribution histogram of Linear Discriminate Analysis (LDA) values (Fig. 2J, K). There were 14 discriminative taxa in the CONN group, with *Romboutsia* LDA scoring the highest. The VPA group was characterized by 26 dominant biomarkers, most notably *Lactobacillus murinus*. Exercise intervention in the VPA-EX group induced a unique 5-taxon signature dominated by *Bacteroides acidifaciens*. T-test analysis revealed significant microbial variations between phylum and genus taxonomic levels. The abundance of *p_Actinobacteria* was significantly elevated in the VPA group compared to CONN and VPA-EX groups (FDR-corrected *P* < 0.05) (Supplementary Fig. 2). In the VPA group, the relative abundance of *g_Alloprevotella* and *g_Rodentibacter* was significantly lower compared to the CONN group (FDR-corrected *P* < 0.05); while the relative abundance of five microorganisms, including *g_unidentified_Coriobacteriales*, was significantly higher. In the VPA-EX group, the relative abundance of seven microorganisms, including *g_Romboutsia*, was significantly lower compared to the VPA group; and the relative abundance of *g_Romboutsia* and *g_Negativibacillus* was significantly lower compared to the CONN group (Supplementary Fig. 3). These findings collectively indicate that voluntary exercise may ameliorate ASD-associated microbial dysbiosis and enhance ecological diversity through exercise-induced restructuring of gut microbiota composition.

### Voluntary wheel running exercise increased SCFAs levels in VPA-induced ASD-like rats

**Fig. 3:**
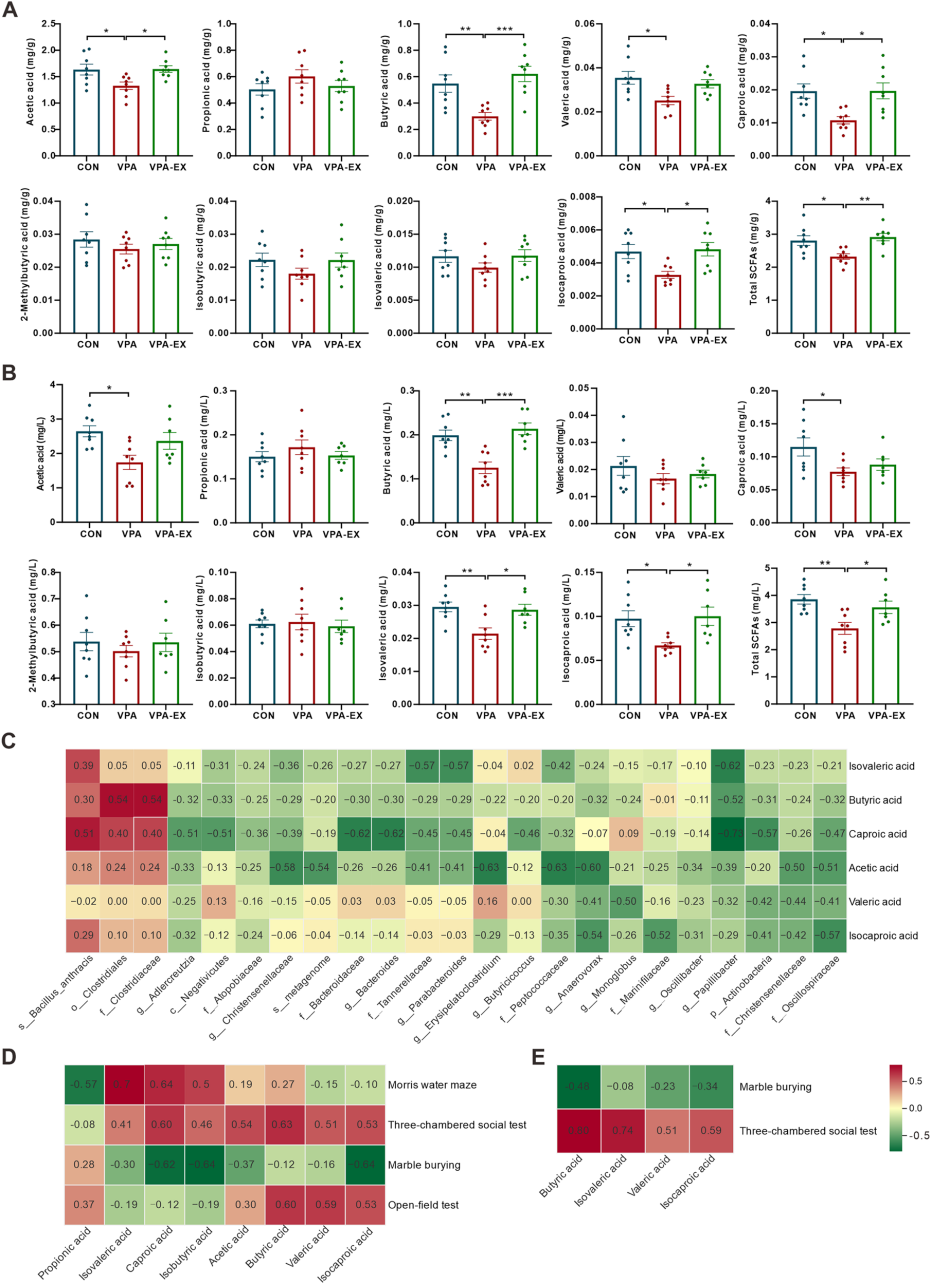
Voluntary wheel running exercise affected the concentration of SCFAs in VPA-induced ASD-like rats. **A** The concentration of SCFAs in feces. **B** The concentration of SCFAs in plasma. **C** Heatmap of Spearman correlation analysis between differential SCFAs and gut microbiota. Heatmap of Spearman correlation analysis between ASD-like behaviors and differential SCFAs in feces (**D**) and plasma (**E**). Red represents positive correlation and green represents negative correlation; the intensity of the color indicates the degree of correlation. The term *s_metagenome* denotes the predicted metagenomic functional profiles of the microbial communities, as inferred by PICRUSt2 analysis. ^*^P < 0.05, ^**^P < 0.01, ^***^P < 0.001, ^****^P < 0.0001.

Quantitative GC-MS analysis revealed exercise-mediated modulation of SCFAs profiles in fecal and plasma. Fecal SCFAs quantification (Fig. 3A) demonstrated that VPA rats exhibited marked reductions in acetic acid, butyric acid, valeric acid, caproic acid, isocaproic acid, and total SCFAs compared to the CONN group. Six-week exercise intervention in VPA-EX rats restored fecal acetic acid, butyric acid, caproic acid, isocaproic acid, and total SCFAs to comparable levels to those of CONN. Plasma SCFA profiling (Fig. 3B) revealed that VPA-induced ASD-like rats displayed decreased acetic acid, butyric acid, caproic acid, isovaleric acid, isocaproic acid, and total SCFAs compared to CONN. Exercise intervention significantly elevated plasma butyric acid, isovaleric acid, isocaproic acid, and total SCFAs in VPA-EX rats.

We analyzed the correlation between the differential SCFAs level in the feces and the relative abundance of the dominantly changed gut microbiota using Spearman correlation analysis (Fig. 3C). The result was displayed in the form of a heatmap, which indicated that acetic acid levels inversely correlated with *Christensenellaceae*, *Oscillospiraceae*, *Anaerovorax*, and *unidentified_Christensenellaceae*. Butyric acid exhibited positive correlations with *Clostridiaceae* and *Clostridiales*, but inverse associations with *Papillibacter*. Caproic acid and isocaproic acid shared negative correlations with *Oscillospiraceae*, and *Actinobacteria*, while valeric acid inversely tracked with *Christensenellaceae*, *Oscillospiraceae*, and *Actinobacteria*. These results demonstrate a close association between exercise-induced gut microbiota alterations and SCFA levels.

Spearman correlation analysis was also performed to further elucidate the relationships between differential SCFAs levels in feces/plasma and ASD-like behaviors. Figure 3D illustrates significant correlations between fecal SCFA levels and ASD-like behaviors. The number of buried marbles in the marble burying test exhibited strong inverse associations with caproic acid and isocaproic acid. Target quadrant duration in the Morris water maze test showed robust positive correlations with caproic acid and isovaleric acid. The central zone exploration time in the open-field test was positively associated with butyric acid and valeric acid. The duration of contact with stranger rats in the three-chambered social test was positively correlated with fecal butyric acid and caproic acid, suggesting a potential role of specific SCFAs in social behavioral in VPA rats. Correlation analyses between plasma SCFA levels and ASD-like behaviors (Fig. 3E) revealed that the number of buried marbles exhibited a negative correlation trend with butyric acid. Strikingly, the duration of contact with stranger rats demonstrated significant positive correlations with butyric acid, isocaproic acid, and isovaleric acid.

### Voluntary wheel running exercise modulated neuroactive substance levels in the hippocampus and prefrontal cortex of VPA-induced ASD-like rats

**Fig. 4:**
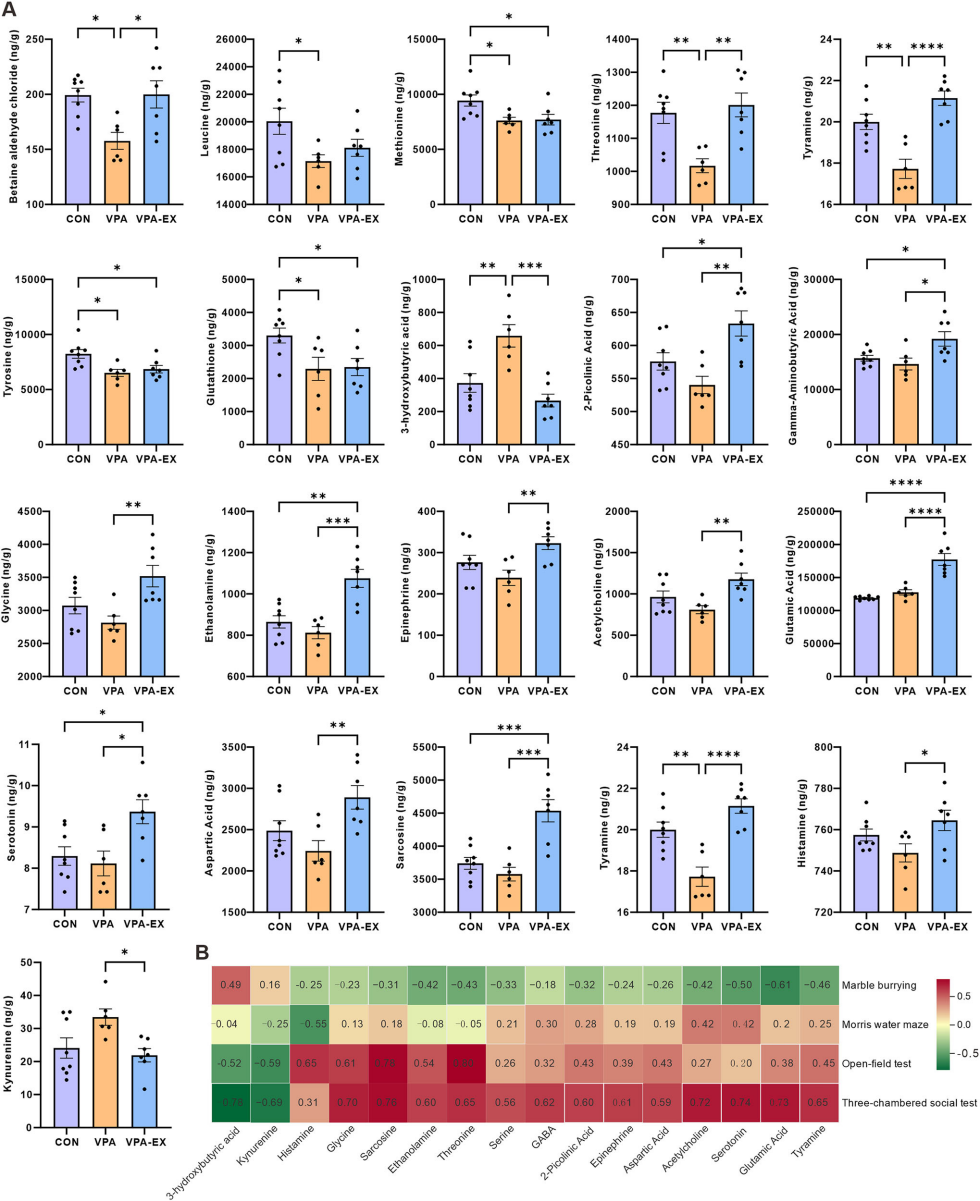
Exercise affected the concentration of neuroactive substances in the hippocampus of VPA-induced ASD-like rats. **A** Results of differential neuroactive substance levels. **B** Heatmap of Spearman correlation analysis between differential neuroactive substances in the hippocampus and ASD-like behaviors. Red represents positive correlation, and green represents negative correlation, the intensity of the color indicates the degree of correlation. ^*^P < 0.05, ^**^P < 0.01^, ***^P < 0.001, ^****^P < 0.0001.

Quantitative analysis of 55 neuroactive substances in the hippocampus and prefrontal cortex (PFC) was performed using liquid chromatography-mass spectrometry (LC-MS). In the hippocampal tissues (Fig. 4A), 37 neuroactive substances were detected. Compared to the CONN group, the VPA group exhibited significant reductions in betaine aldehyde chloride, leucine, methionine, threonine, tyramine, tyrosine, and glutathione, alongside a marked elevation in 3-hydroxybutyric acid. Six-week voluntary exercise intervention significantly modulated the homeostasis of neuroactive substances. VPA-EX group showed increased levels of 2-picolinic acid, γ-aminobutyric acid, glycine, ethanolamine, epinephrine, acetylcholine, betaine aldehyde chloride, glutamic acid, serotonin, aspartic acid, sarcosine, threonine, tyramine, and histamine compared to VPA counterparts. Conversely, 3-hydroxybutyric acid and kynurenine concentrations were significantly attenuated following exercise. VPA-EX group maintained elevated 2-picolinic acid, γ-aminobutyric acid, ethanolamine, glutamic acid, serotonin, and sarcosine relative to CONN group, while showing persistent reductions in methionine, tyrosine, and glutathione.

Spearman correlation analysis revealed significant relationships between hippocampal neuroactive substances levels and ASD-like behaviors (Fig. 4B). The number of buried marbles in the marble burying test showed a significant negative correlation with glutamic acid levels, suggesting reduced glutamatergic signaling may underlie increased stereotypic behaviors in VPA rats. The central zone exploration time in the open-field test demonstrated strong positive correlations with glycine, sarcosine, histamine, and threonine, while showing a negative trend with kynurenine. The duration of contact with stranger rats in the three-chambered social test exhibited positive associations with γ-aminobutyric acid, serotonin, acetylcholine, glycine, sarcosine, epinephrine, threonine, glutamic acid, and tyramine, while negative associations with 3-hydroxybutyric acid and kynurenine.

**Fig. 5:**
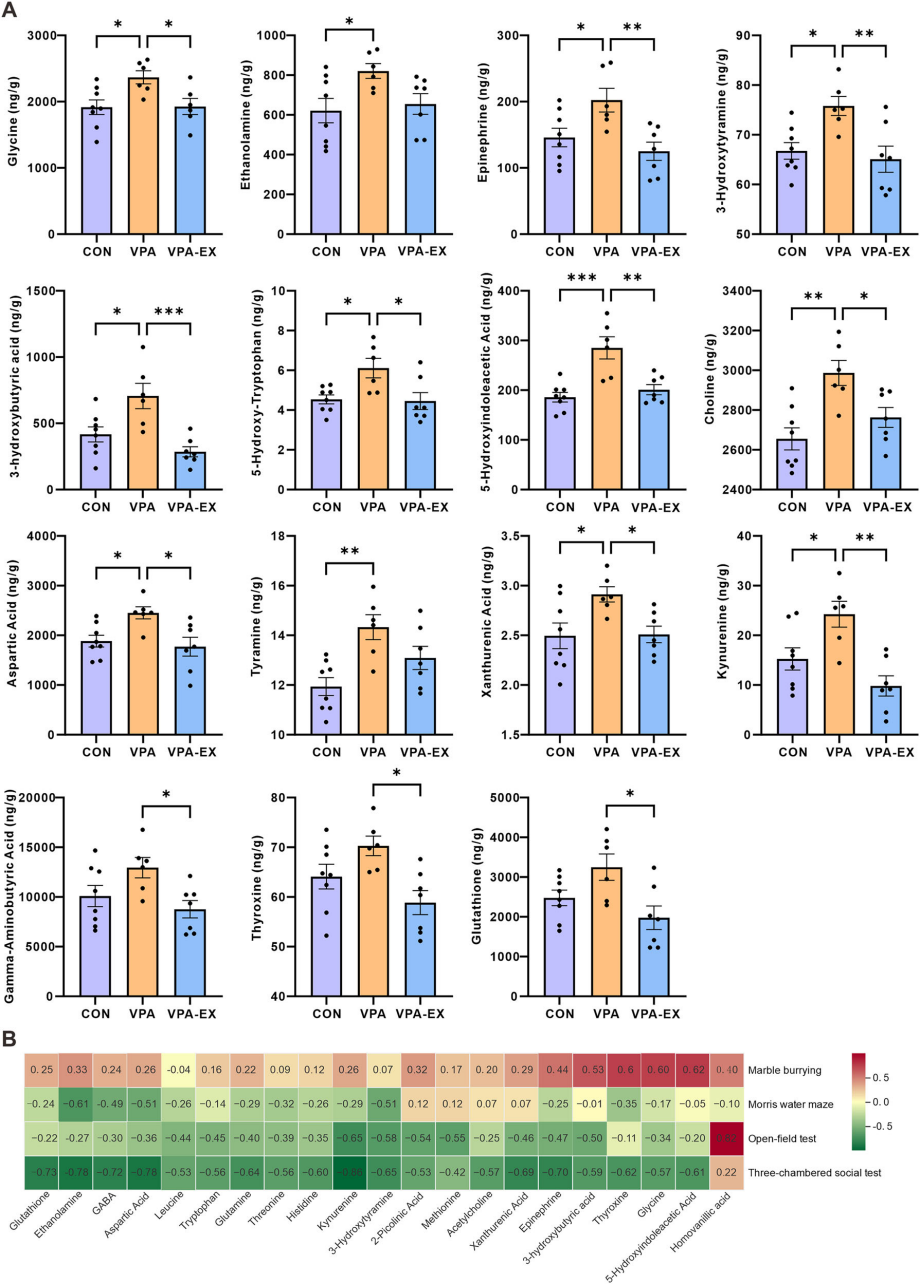
Exercise affected the concentration of neuroactive substances in the PFC of VPA-induced ASD-like rats. **A** Results of differential neuroactive substance levels. **B** Heatmap of Spearman correlation analysis between differential neuroactive substances in the PFC and ASD-like behaviors. Red represents positive correlation, and green represents negative correlation, the intensity of the color indicates the degree of correlation. ^*^P < 0.05, ^**^P < 0.01^, ***^P < 0.001, ^****^P < 0.0001.

LC-MS quantification identified 38 neuroactive substances in the PFC (Fig. 5A). Compared to the CONN group, the VPA group exhibited significant elevations in glycine, ethanolamine, epinephrine, dopamine (3-hydroxytyramine), 3-hydroxybutyric acid, 5-hydroxy-tryptophan, 5-hydroxyindoleacetic acid, choline, aspartic acid, tyramine, xanthurenic acid, and kynurenine. The exercise intervention induced striking normalization of prefrontal neuroactive substances profiles. VPA-EX group showed significant reductions versus VPA counterparts in γ-aminobutyric acid, glycine, epinephrine, dopamine, 3-hydroxybutyric acid, 5-hydroxy-tryptophan, 5-hydroxyindoleacetic acid, choline, aspartic acid, thyroxine, xanthurenic acid, kynurenine, and glutathione. Collectively, these findings in the hippocampus and PFC demonstrate that voluntary exercise effectively restores neuroactive substances homeostasis in key brain regions associated with ASD pathophysiology.

Spearman correlation analysis was performed to elucidate the relationships between prefrontal neuroactive substance levels and ASD-like behaviors (Fig. 5B). Stereotypic behaviors (the number of buried marbles) showed significant positive correlations with 5-hydroxyindoleacetic acid, glycine, and thyroxine. Spatial memory performance (target quadrant duration) in the Morris water maze test negatively correlated with ethanolamine levels. The exploratory behaviors (central zone duration) in the open-field test demonstrated a strong inverse relationship with kynurenine concentrations. Social interaction with novel rats in the three-chamber social test was associated with elevated levels of γ-aminobutyric acid, 5-hydroxyindoleacetic acid, dopamine, aspartic acid, kynurenine, thyroxine, epinephrine, ethanolamine, glutathione, and xanthurenic acid.

### Voluntary wheel running exercise ameliorated neuroinflammation in the hippocampus and PFC of VPA-induced ASD-like rats

**Fig. 6:**
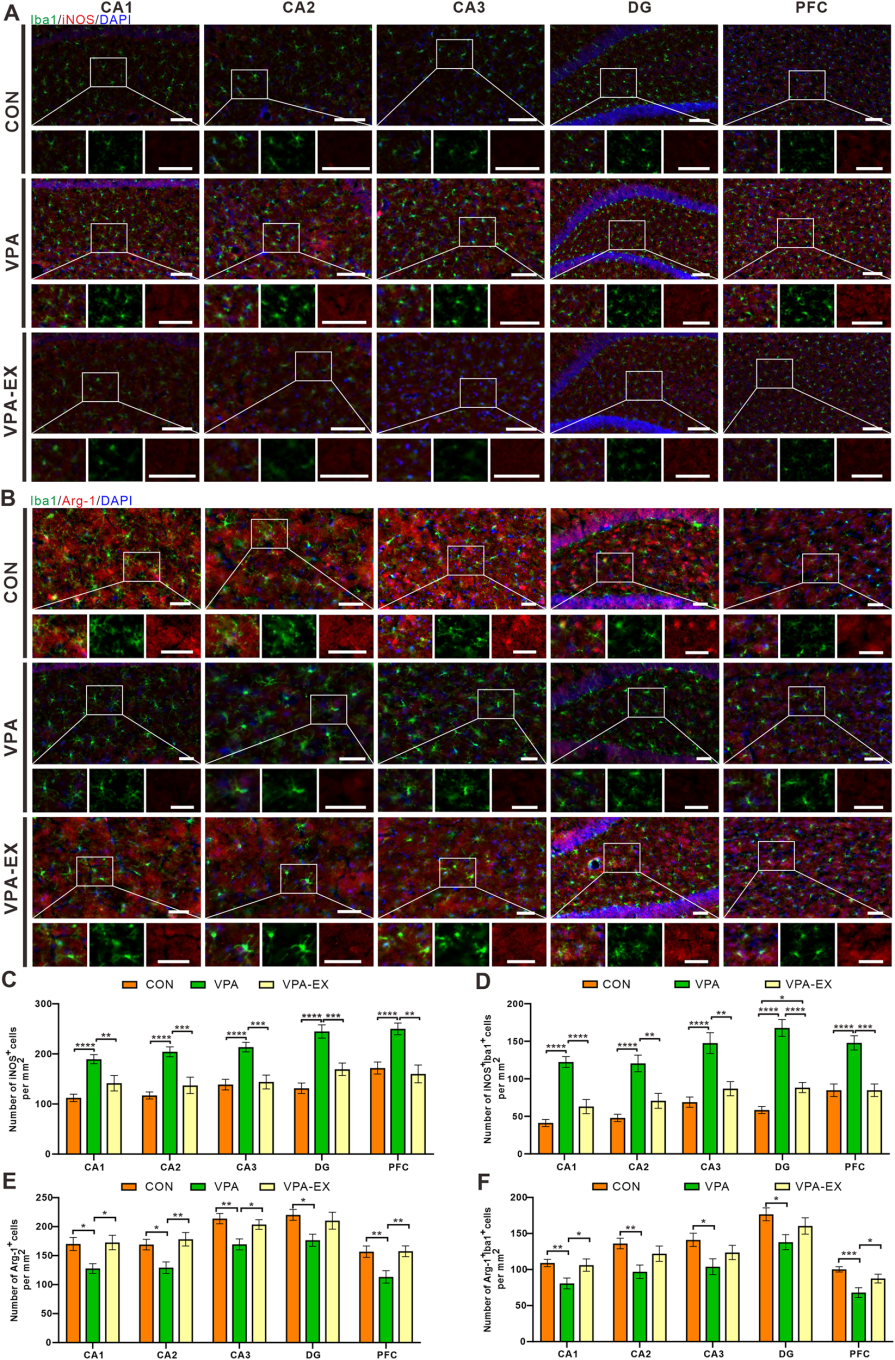
Exercise inhibited microglia reactivity and modulated M1/M2 microglia polarization in VPA-induced ASD-like rats. **A** Representative images of double-staining immunofluorescence for Iba1 (green) and iNOS (red) with DAPI nuclear counterstain in the cornu ammonis 1 (CA1), cornu ammonis 2 (CA2), cornu ammonis 3 (CA3) subregions, and dentate gyrus (DG) of the hippocampus, as well as in the PFC. **B** Representative images of double-staining immunofluorescence for Iba1 (green) and Arg-1 (red) with DAPI nuclear counterstain in the CA1, CA2, CA3 subregions and DG of the hippocampus, as well as in the PFC. **C** The number of iNOS ^+^ cells per mm^2^. **D** The number of iNOS^+^Iba1^+^ cells per mm^2^. **E** The number of Arg-1^+^ cells per mm^2^. **F** The number of Arg-1^+^Iba1^+^ cells per mm^2^. Data were expressed as mean ± SEM. Scale bar = 100 μm, ^*^P < 0.05, ^**^P < 0.01^, ***^P < 0.001 and ^****^P < 0.0001. All experiments were performed in triplicate, four animals from each group, and twelve randomly selected images.

To investigate the effects of exercise on microglial dynamics, we performed immunofluorescence staining for ionized calcium-binding adapter molecule 1 (Iba1) and assessed pro-inflammatory/anti-inflammatory (M1/M2) polarization state through co-localization with inducible nitric oxide synthase (iNOS) and arginase-1 (Arg-1), respectively (Fig. 6A, B). Results of immunofluorescence staining showed that VPA rats exhibited significantly a significant increase in the number of Iba1^+^ cells compared to the CONN group, indicating pathological microglial reactivity. Voluntary wheel running exercise intervention (VPA-EX group) effectively attenuated this reactivity, with a corresponding reduction in the number of Iba1^+^ cells in the respective brain regions compared to the VPA counterparts (Supplementary Fig. 4). Quantitative analysis of immunofluorescence co-localization revealed a pronounced M1-polarization bias in VPA animals. VPA rats exhibited a significant elevation of iNOS^+^/Iba1^+^ co-localized cells in the hippocampus and PFC compared to the CONN group. Voluntary exercise intervention (VPA-EX group) markedly attenuated this pro-inflammatory polarization, reducing the number of iNOS^+^/Iba1^+^ cells in the respective brain regions compared to VPA counterparts (Fig. 6C, D). Conversely, VPA rats showed a pronounced depletion of Arg-1^+^/Iba1^+^ co-localized cells in the hippocampus and PFC relative to the CONN group. Exercise intervention reversed this deficit, increasing the number of Arg-1^+^/Iba1^+^ cells in the corresponding regions compared to the VPA group (Fig. 6E, F). These results suggested that voluntary exercise ameliorated microglia reactivity and modulated M1/M2 microglia polarization in the hippocampus and PFC of VPA rats.

To comprehensively assess neuroinflammatory and neuropathological characteristics, we further evaluated astrocyte reactivity and neuronal density using glial fibrillary acidic protein (GFAP) and neuronal nuclei (NeuN) immunofluorescence, respectively (Supplementary Fig. 5A, B). Quantitative analysis revealed increased GFAP immunoreactivity in VPA rats, with a significant increase in the number of GFAP^+^ cells in the hippocampus and PFC compared to the CONN group. Voluntary exercise intervention (VPA-EX group) attenuated this pathological reactivity, reducing the number of GFAP^+^ cells in the respective regions versus VPA counterparts (Supplementary Fig. 5C). NeuN immunofluorescence analysis revealed significant neuronal alterations across hippocampal subregions and PFC. Compared to CONN controls, VPA rats exhibited reduced mean fluorescence intensity of NeuN in hippocampal CA1, CA2, DG, and PFC, suggesting neuronal loss or compromised maturation in ASD pathophysiology. Following six weeks of voluntary exercise intervention, VPA-EX rats exhibited region-specific restoration of NeuN levels, demonstrating an increase in DG and PFC relative to VPA counterparts (Supplementary Fig. 5D). These findings collectively suggest that exercise exerts selective neuroprotective effects, enhancing neuronal integrity in ASD-vulnerable regions, which are critical for cognitive and social functions.

To explore the correlation between central neuroinflammatory responses and ASD-like behaviors, we used Mantel test analysis to examine the correlation between the expression levels of inflammatory markers (Iba1, iNOS, Arg-1, GFAP) and neuronal markers (NeuN) in the hippocampal subregions (CA1, CA2, CA3, DG) and PFC, and ASD-like behavioral indicators (Supplementary Fig. 6). In each subregion of the hippocampus, GFAP expression exhibited robust positive correlations with Iba1 and iNOS, while inversely correlating with Arg-1 levels. Similarly, Iba1 was positively correlated with iNOS and negatively correlated with Arg-1. In the PFC, GFAP was positively correlated with Iba1 and negatively correlated with Arg-1, while Iba1 again associated with iNOS upregulation and Arg-1 suppression. Crucially, correlation analysis between ASD-like behaviors and neuroinflammatory markers revealed that elevated iNOS levels in the hippocampus and PFC was negatively correlated with social interaction time with unfamiliar rats in the three-chamber social test. Additionally, NeuN expression was positively correlated with spatial memory performance (target quadrant duration) in the Morris water maze test.

### Correlations analysis of differential SCFAs, neuroactive substances and neuroinflammation

**Fig. 7:**
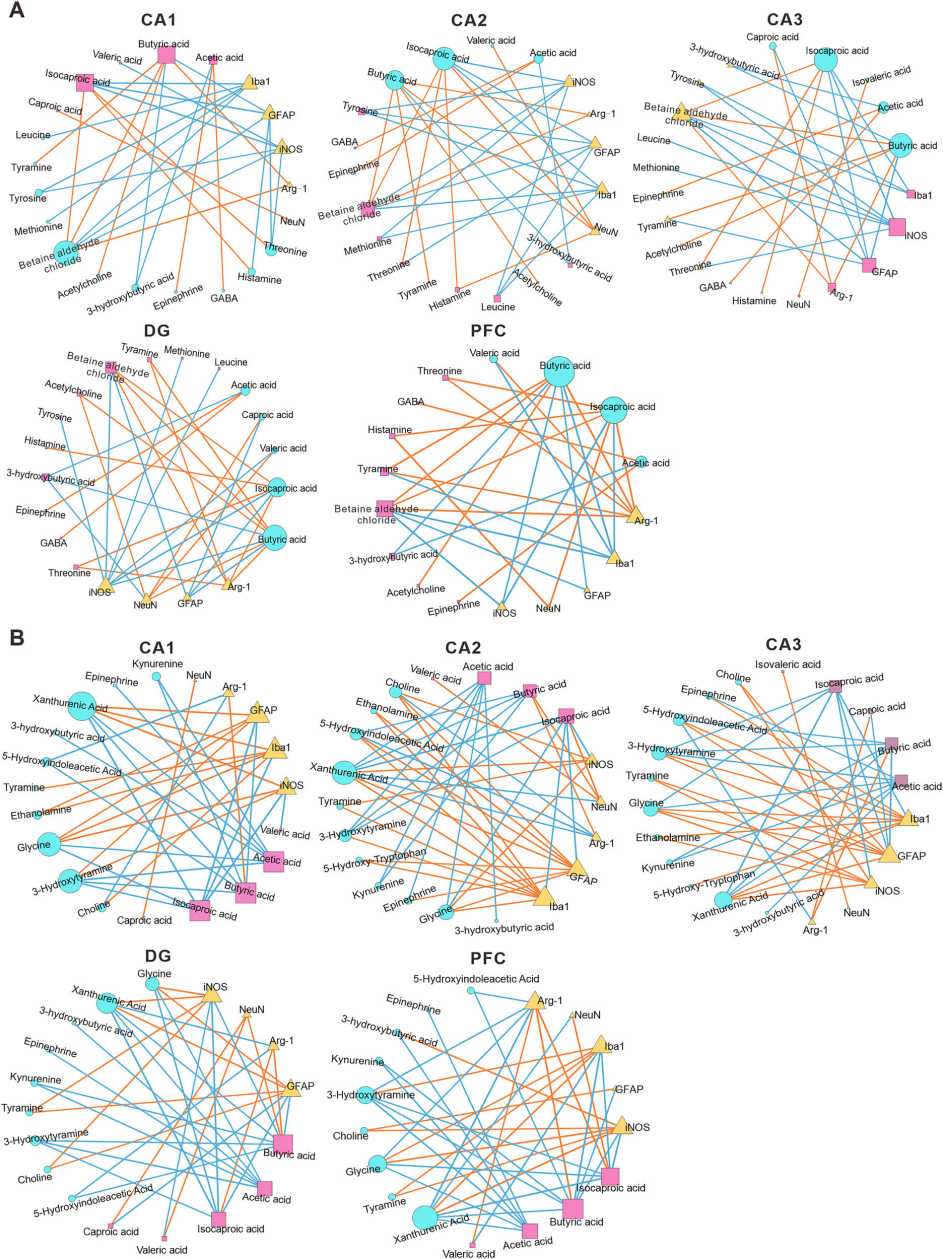
Results of the Pearson correlation network analysis. An analysis of the Pearson correlation network between the differential SCFAs, neuroactive substances in the hippocampus (**A**) and PFC (**B**), and neuroinflammation. The node size represents connectivity (the number of substances significantly correlated with the substance), with larger nodes indicating higher connectivity. The Diverse line colors between nodes represent positive (yellow) and negative (blue) correlation coefficients. Line thickness indicates the strength of the correlation, with thicker lines representing stronger correlations.

Pearson correlation network analysis revealed a complex interplay between the differential SCFAs, neuroactive substances, and neuroinflammation (Fig. 7). The analysis revealed that the levels of butyric acid, caproic acid, and isocaproic acid, which altered significantly in VPA rats, exhibited robust negative correlations with neuroinflammatory markers (GFAP, Iba1, iNOS), while positively associating with anti-inflammatory Arg-1 and neuronal marker NeuN. Notably, acetic acid, butyric acid, and caproic acid further demonstrated extensive connectivity with hippocampal and prefrontal neuroactive substances, suggesting bidirectional gut-brain crosstalk. In the hippocampus, differential neuroactive substances such as betaine aldehyde chloride, leucine, methionine, threonine, tyrosine, tyramine, and histamine were negatively correlated with GFAP, Iba1, and iNOS expression, while positively correlated with Arg-1. In the PFC, differential neuroactive substances such as glycine, ethanolamine, tyramine, 3-hydroxytyramine, choline, xanthurenic acid, 5-hydroxy-tryptophan, and 5-hydroxyindoleacetic acid were positively correlated with GFAP, Iba1, and iNOS expression but negatively correlated with Arg-1. These spatially divergent networks imply that gut-derived SCFAs may attenuate neuroinflammation by suppressing glial reactivity and enhancing Arg-1-mediated anti-inflammatory responses, while differentially modulating region-specific neurotransmitter circuits. The coordinated yet heterogeneous interactions among SCFAs, neuroactive substances, and neuroinflammation underscore a multifaceted gut-brain axis mechanism potentially driving ASD-related behavioral and cognitive deficits.

### Vagotomy attenuates voluntary wheel running exercise-induced amelioration of VPA-induced ASD-like behaviors

**Fig. 8:**
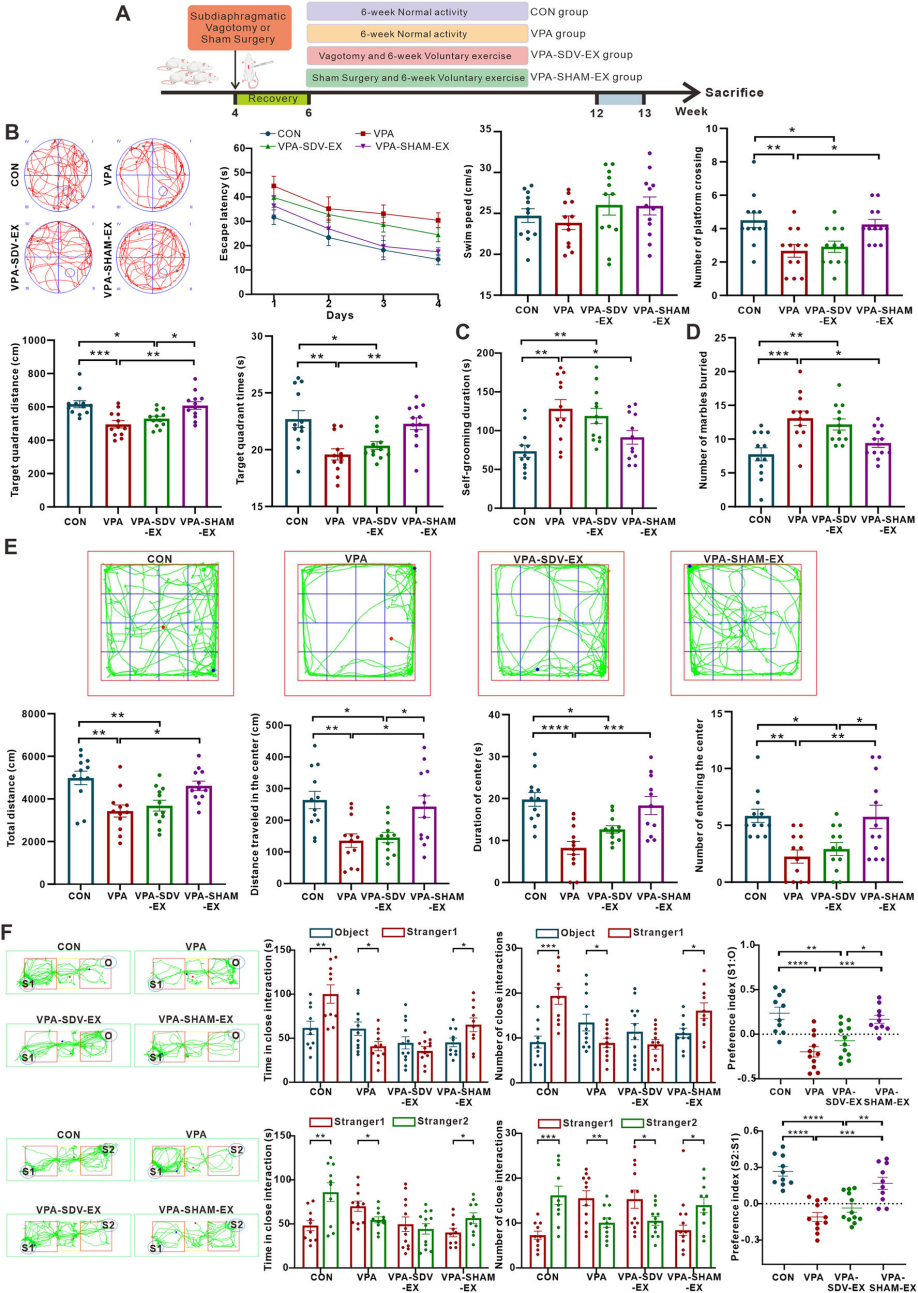
Effects of voluntary running wheel exercise following vagotomy on ASD-like behaviors. **A** Timeline of the experimental procedure. **B** The escape latency in the learning phase, the swim speed, the number of exact crossings over the previously hidden platform in the probe phase, and the distance and time in the target quadrant in the probe phase in the Morris water maze test. **C** Duration in self-grooming behavior. **D** The number of buried marbles. **E** The total distance traveled, average speed, distance traveled in the center, time spent in the center, average speed of center, and the number of entering the center in the open-field test. **F** The time and number of times spent with object (O), stranger 1 (S1), and stranger 2 (S2), and the preference indexes in the three-chamber social test. ^*^P < 0.05, ^**^P < 0.01^, ***^P < 0.001, ^****^P < 0.0001.

To determine the role of the gut-brain axis vagal pathway in the improvement of ASD-like behaviors by voluntary running wheel exercise, we performed subdiaphragmatic vagotomy on VPA-induced ASD model rats. After 6 weeks of voluntary running wheel exercise intervention, behavioral assessments were conducted (Fig. 8A). Notably, vagotomy did not affect the volume of voluntary running exercise, nor were there differences in body weight between the critical experimental groups, ruling out these potential confounding factors (Supplementary Fig. 7).

Morris water maze tests revealed (Fig. 8B) that vagotomized VPA rats subjected to 6 weeks of voluntary wheel running exercise (VPA-SDV-EX group) exhibited significantly prolonged escape latency compared to the CONN group, along with reduced platform crossings, decreased distance in the target quadrant, and diminished time spent in the target quadrant. These parameters showed no statistical difference from the VPA group, suggesting vagotomy may impair exercise-mediated improvements in spatial learning and navigational memory in ASD-like rats. In contrast, VPA-SHAM-EX group rats that underwent exercise intervention after sham surgery demonstrated enhanced cognitive performance with shortened escape latency, increased platform crossings, greater distance in the target quadrant, and prolonged duration in the target quadrant. These findings collectively indicate the vagus nerve plays an important modulatory role in mediating exercise-induced cognitive enhancement in ASD-like behaviors.

Self-grooming analysis (Fig. 8C) and marble burying tests (Fig. 8D) demonstrated that the VPA-SDV-EX rats displayed significantly exacerbated self-grooming duration and number of buried marbles compared to the CONN group. Conversely, VPA-SHAM-EX animals exhibited marked reductions in self-grooming duration and buried marbles, achieving behavioral profiles comparable to CONN rats. These findings suggest that while exercise intervention effectively ameliorates repetitive/stereotypic behaviors in ASD-like models, vagal integrity appears essential for mediating these benefits.

In the open-field test (Fig. 8E, Supplementary Fig. 8), VPA-SDV-EX rats exhibited marked reductions in total distance traveled, average speed, distance of center, duration of center, and number of entering the center compared to the CONN group. Conversely, VPA-SHAM-EX rats demonstrated robust increases in total distance, average speed, distance of center, duration of center, and number of entering the center relative to the VPA group. These data collectively highlight vagal integrity as a key factor in exercise-induced augmentation of spontaneous activity and environmental exploration in VPA rats.

In the sociability test (Fig. 8F), VPA-SDV-EX rats showed no significant preference for interaction time between novel conspecifics (Stranger 1) and inanimate objects. Similarly, VPA-SDV-EX animals exhibited significantly reduced social preference indices for Stranger 1 compared to CONN and VPA-SHAM-EX groups. In contrast, VPA-SHAM-EX rats demonstrated enhanced sociability with increased interaction time toward Stranger 1, accompanied by elevated social preference indices compared to the VPA group. In the social novelty test, VPA-SHAM-EX rats displayed diminished social novelty responses, evidenced by reduced interaction time toward Stranger 2 and significantly lower social preference indices compared to CONN and VPA-SHAM-EX groups. Conversely, VPA-SHAM-EX animals exhibited augmented social novelty with increased sniffing duration, sniffing bout frequency, and social preference indices for Stranger 2 relative to the VPA group.

### Voluntary wheel running exercise following vagotomy affected the composition of gut microbiota in VPA-induced ASD-like rats

**Fig. 9:**
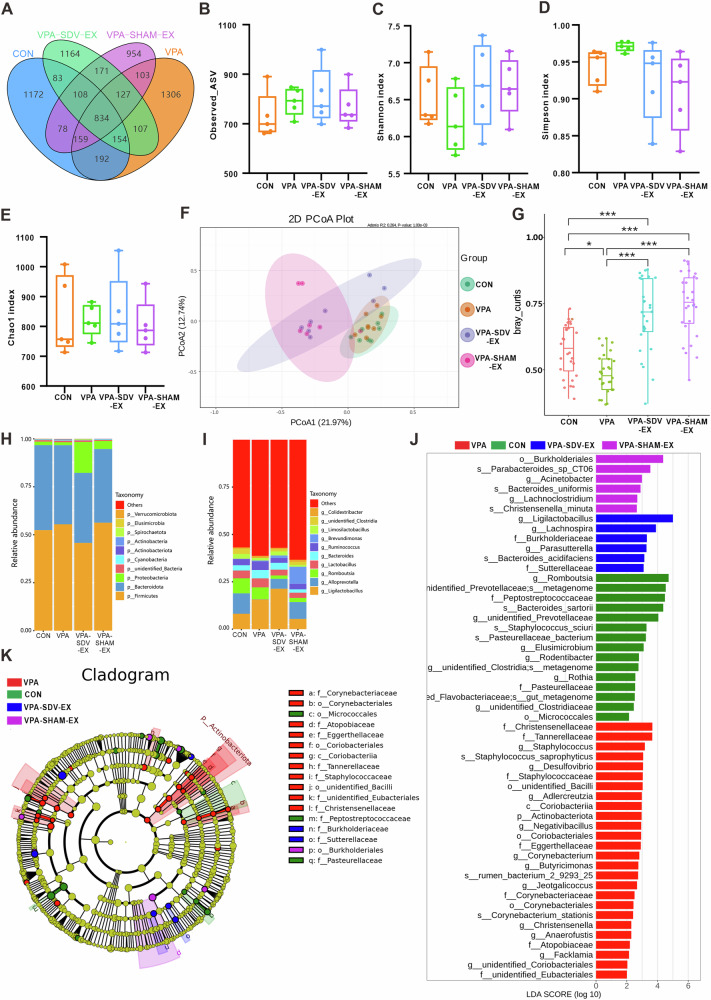
Voluntary wheel running exercise following vagotomy affected the composition of gut microbiota in VPA-induced ASD-like rats. **A** Venn diagram illustrates the numbers of ASVs in the CONN, VPA, VPA-SDV-EX, and VPA-SHAM-EX groups. Alpha diversity is illustrated using a box plot of the **B** Observed_ASV, **C** Shannon, **D** Simpson, and **E** Chao1 indices. **F** Beta-diversity was assessed using PCoA. **G** The differences in β diversity among groups were analyzed using Tukey’s test. The top 10 relative abundances of gut microbiota at the phylum (**H**), and genus **I** taxonomic levels for the four groups. **J** Histogram of LDA scores for bacterial taxa significantly enriched in gut microbiota from each group (LDA score >2). The LDA score indicates the effect size and ranking of each differentially abundant taxon. **K** Cladogram from the LEfSe illustrating showing differences in fecal taxa. ^*^P < 0.05, ^**^P < 0.01, ^***^P < 0.001.

Venn diagram (Fig. 9A) revealed that the CONN, VPA, VPA-SDV-EX, and VPA-SHAM-EX groups harbored 1172, 1306, 1164, and 954 unique ASVs respectively, while sharing a conserved core microbiota of 834 ASVs. Alpha diversity analysis (Fig. 9B–E) demonstrated no statistically significant differences in gut microbiota alpha diversity indices across experimental groups, indicating that voluntary exercise did not induce significant alterations in the richness or diversity of intestinal microbial communities in VPA rats, irrespective of subdiaphragmatic vagotomy. PCoA results (Fig. 9F) revealed clear spatial separation between VPA-SDV-EX, VPA-SHAM-EX, and VPA groups, confirming significant inter-group structural divergence in gut microbial communities. VPA-SDV-EX and VPA-SHAM-EX groups exhibited greater intra-group dispersion than VPA group, suggesting exercise intervention amplifies β-diversity heterogeneity in VPA subjects. Further analysis (Fig. 9G) demonstrated significant beta diversity differences between the VPA group and the CONN, VPA-SDV-EX, and VPA-SHAM-EX groups. These findings suggest that exercise intervention after vagotomy may still positively impact the diversity of the gut microbiota in ASD-like rats.

Taxonomic profiling at the phylum level (Fig. 9H) revealed the dominant phyla in all four groups of rats were *Firmicutes*, *Bacteroidota*, and *Proteobacteria*. At the genus level (Fig. 9I), CONN rats exhibited predominant colonization by *Alloprevotella*, *Ligilactobacillus*, and *Romboutsia*, while the VPA group showed significant *Ligilactobacillus* overgrowth accompanied by reduced *Alloprevotella* and *Romboutsia*. In the VPA-SDV-EX group, the dominant genera were *Ligilactobacillus*, *Alloprevotella*, and *Bacteroides*. In the VPA-SHAM-EX group, they were *Ligilactobacillus*, *Alloprevotella*, and *Brevundimonas*.

LDA score (Fig. 9J, K) showed that CONN rats harbored 15 discriminative microbes, including *Romboutsia*, while VPA models exhibited 26 signature species dominated by *Lactobacillus murinus*. Exercise interventions substantially reduced biomarker complexity, with VPA-SDV-EX and VPA-SHAM-EX groups showing 6 discriminative taxa each—*Ligilactobacillus* and *Burkholderiales* serving as respective keystone taxa. Differential abundance analysis revealed that at the phylum level, the abundance of *Actinobacteria* within the VPA group was significantly higher than that in both the VPA-SDV-EX and VPA-SHAM-EX groups (Supplementary Fig. 9). At the genus level, the abundance of *Allobaculum* in the VPA-SHAM-EX group rats was significantly reduced compared to the VPA-SDV-EX group rats. Compared with the VPA group, the VPA-SHAM-EX group was specifically enriched in *Alistipes* and *Ruminococcus* alongside depletion of 16 taxa, including *Ligilactobacillus*, while VPA-SDV-EX rats showed increased *Bacillus* and *Parasutterella* with concurrent decreasing the abundance of 8 microorganisms, including *Romboutsia*. Compared to the CONN group, the VPA-SHAM-EX group had reduced levels of *Romboutsia* and five other microbes, while the VPA-SDV-EX group had decreased levels of *Romboutsia* and four others, but increased levels of *Parasutterella* and *unidentified Christensenellaceae* (Supplementary Fig. 10). These exercise-driven biomarker signatures suggest that both exercise and vagal integrity critically shape gut microbial architecture, with vagotomy influencing rather than abolishing exercise-induced community restructuring.

### Voluntary wheel running exercise following vagotomy affected SCFAs levels in VPA-induced ASD-like rats

**Fig. 10:**
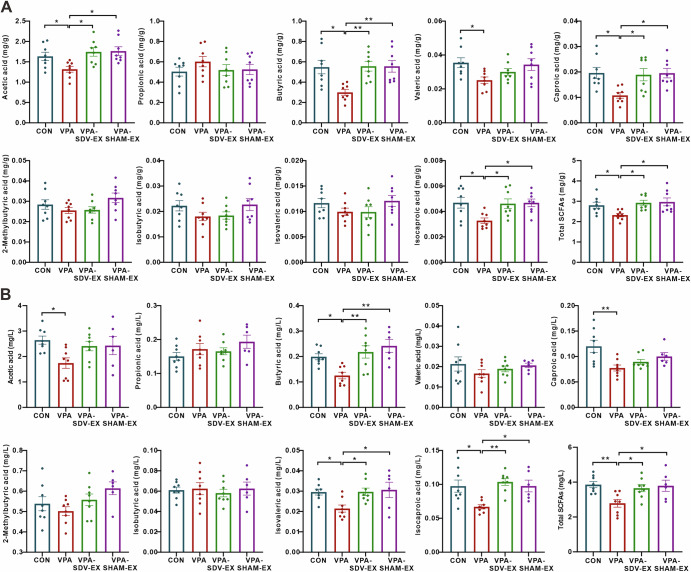
Voluntary wheel running exercise following vagotomy affected the concentration of SCFAs in VPA-induced ASD-like rats. **A** The concentration of SCFAs in feces. **B** The concentration of SCFAs in plasma. ^*^P < 0.05, ^**^P < 0.01^, ***^P < 0.001, ^****^P < 0.0001.

Quantitative analysis of fecal SCFAs revealed significant exercise intervention effects (Fig. 10A). Compared to VPA animals, VPA-SDV-EX and VPA-SHAM-EX groups exhibited elevated fecal concentrations of acetate, butyrate, caproate, isocaproate, and total SCFAs, reaching levels comparable to CONN rats. The analysis of SCFAs in plasma (Fig. 10B) demonstrated congruent patterns, with exercise interventions restoring butyrate, isovalerate, isocaproate, and total SCFAs to physiological concentrations. These findings collectively indicate that exercise-mediated SCFAs restoration may involve vagus-independent pathways, while preserved vagal integrity may optimize microbial metabolite bioavailability, pointing to the possibility of dual mechanisms for gut-derived SCFAs modulation in neurodevelopmental disorders.

### Vagotomy subtly blunted the modulatory effects of voluntary wheel running exercise on neuroactive substances in the hippocampus and PFC of VPA-induced ASD-like rats

**Fig. 11:**
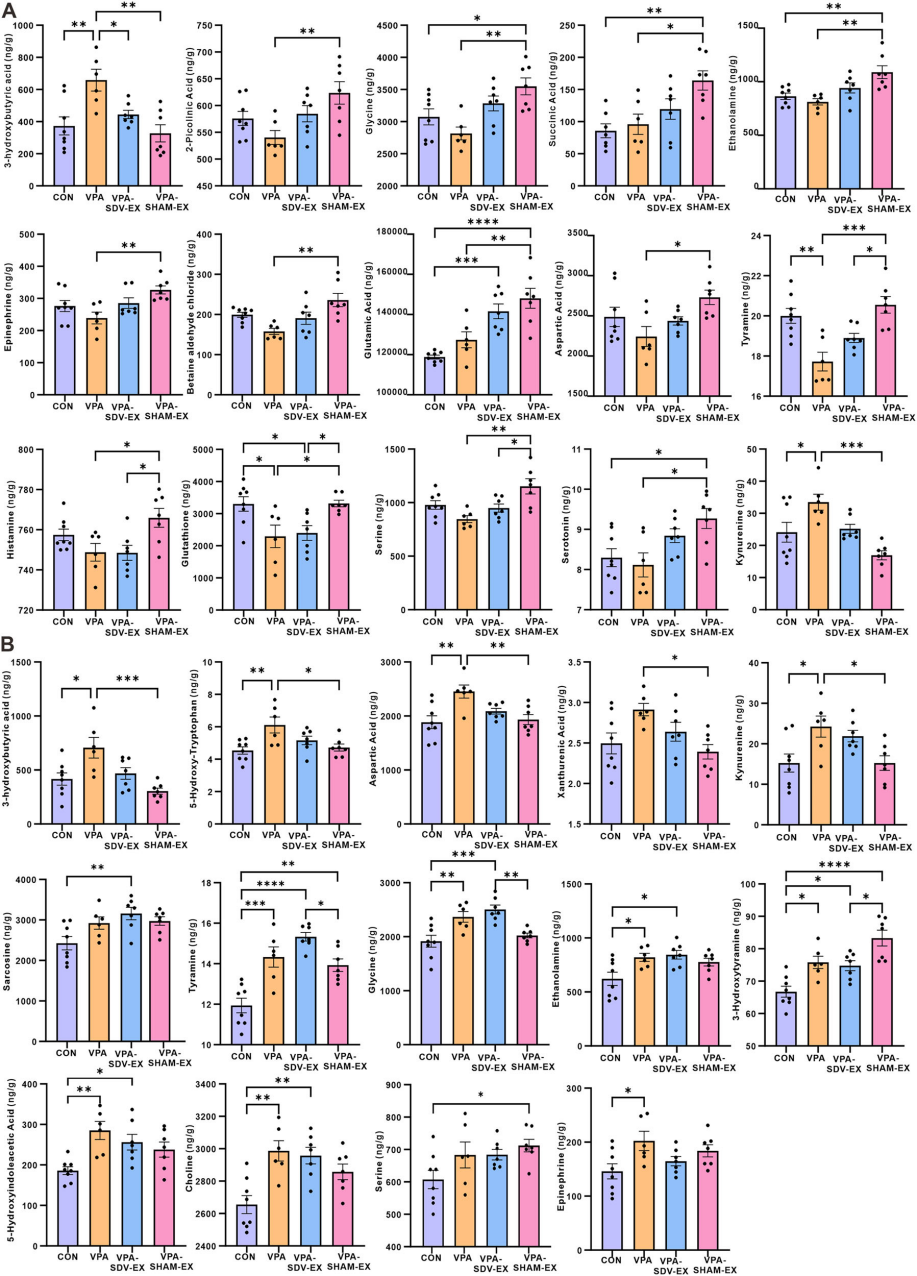
Vagotomy subtly blunted the exercise-induced modulation of neuroactive substance levels in VPA-induced ASD-like rats. **A** Results of differential neuroactive substance levels in the hippocampus. **B** Results of differential neuroactive substance levels in the PFC. ^*^P < 0.05, ^**^P < 0.01^, ***^P < 0.001, ^****^P < 0.0001.

Neurochemical analysis revealed distinct exercise-mediated neuroactive substance regulation patterns dependent on vagal integrity. In the hippocampus (Fig. 11A), VPA-SDV-EX rats showed selective reduction of 3-hydroxybutyric acid versus VPA group, whereas VPA-SHAM-EX animals exhibited broad neuromodulation with significant elevations in 2-picolinic acid, glycine, succinic acid, ethanolamine, epinephrine, betaine aldehyde chloride, glutamic acid, aspartic acid, tyramine, histamine, glutathione, L-serine, and serotonin, alongside reduced 3-hydroxybutyric acid and kynurenine. Compared with the VPA-SDV-EX group, the levels of tyramine, histamine, glutathione, and serine were significantly increased in the VPA-SHAM-EX group. PFC analysis demonstrated blunted exercise regulation in vagotomized rats (Fig. 11B), with VPA-SDV-EX rats showing no significant neuroactive substances differences versus VPA group. Conversely, VPA-SHAM-EX rats exhibited normalized neuromodulation through reduced 3-hydroxybutyrate, 5-hydroxy-tryptophan, aspartate, xanthurenic acid, and kynurenine. Compared to CONN, VPA-SDV-EX rats developed aberrant elevations in sarcosine, tyramine, glycine, ethanolamine, dopamine, 5-hydroxyindoleacetic acid, and choline, VPA-SHAM-EX animals showed increases limited to tyramine, L-serine, and 3-hydroxytyramine. Compared with rats in the VPA-SDV-EX group, VPA-SHAM-EX group exhibited significantly reduced levels of tyramine and glycine, while 3-hydroxytyramine levels were markedly elevated.

### Voluntary wheel running exercise attenuated neuroinflammation in the hippocampus and PFC in a vagus nerve-dependent manner

**Fig. 12:**
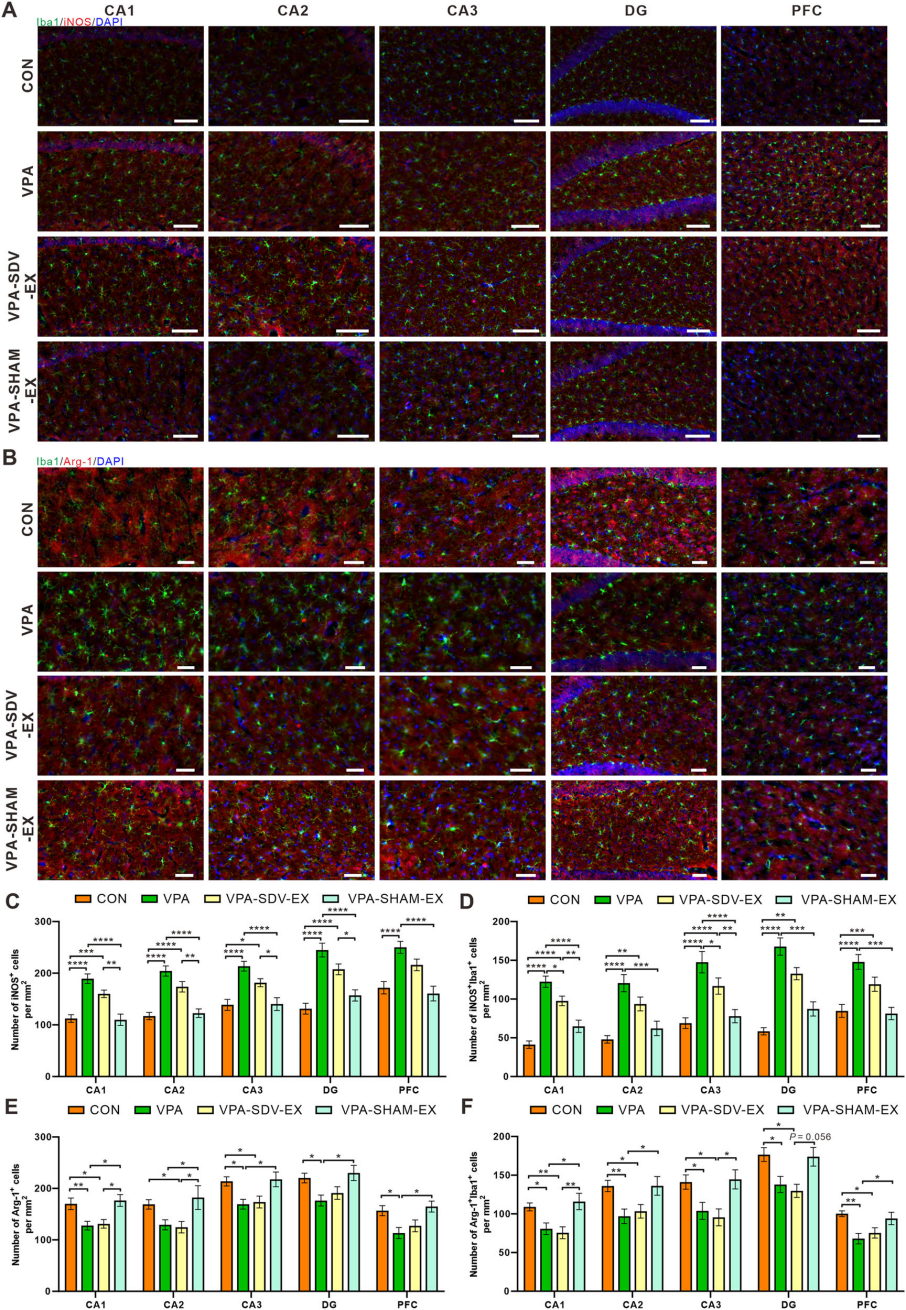
Vagotomy attenuated the modulatory effect of exercise on microglia polarization in VPA-induced ASD-like rats. **A** Representative images of double-staining immunofluorescence for Iba1 (green) and iNOS (red) with DAPI nuclear counterstain in the CA1, CA2, CA3, and DG subregions of the hippocampus, as well as in the PFC. **B** Representative images of double-staining immunofluorescence for Iba1 (green) and Arg-1 (red) with DAPI nuclear counterstain in the CA1, CA2, CA3, and DG subregions of the hippocampus, as well as in the PFC. **C** The number of iNOS^+^ cells per mm^2^. **D** The number of iNOS^+^Iba1^+^ cells per mm^2^. **E** The number of Arg-1^+^ cells per mm^2^. **F** The number of Arg-1^+^Iba1^+^ cells per mm^2^. Data were expressed as mean ± SEM. Scale bar = 100 μm, ^*^P < 0.05, ^**^P < 0.01^, ***^P < 0.001 and ^****^P < 0.0001. All experiments were performed in triplicate, four animals from each group, and twelve randomly selected images.

Immunofluorescence analysis of Iba1 expression revealed that exercise regulation of microglia dependent on vagal integrity (Supplementary Fig. 11A). Compared to VPA-SDV-EX rats, VPA-SHAM-EX animals exhibited significantly reduced numbers of Iba1^+^ cells in the hippocampus and PFC (Supplementary Fig. 11B). Co-localization studies demonstrated that the regulation of microglia polarization by exercise was dependent on the vagus nerve (Fig. 12A–F). Compared with the VPA group, VPA-SHAM-EX rats exhibited significant reductions in pro-inflammatory iNOS⁺Iba1⁺ cells in the hippocampus and PFC, whereas VPA-SDV-EX animals showed no significant changes (Fig. 12C, D). Conversely, VPA-SHAM-EX group showed increased anti-inflammatory Arg-1⁺Iba1⁺ cell populations, an effect that was absent in VPA-SDV-EX rats (Fig. 12E, F).

GFAP immunofluorescence quantification revealed region-specific exercise effects on astrocytic reactivity (Supplementary Fig. 12A, C). Compared to the VPA group, VPA-SDV-EX rats exhibited comparable numbers of GFAP^+^ cells in the hippocampus and PFC. In contrast, VPA-SHAM-EX animals demonstrated a significant reduction in GFAP^+^ cells, indicating vagal integrity is required for exercise-mediated attenuation of astrocytic reactivity. NeuN immunofluorescence demonstrated neuroprotective effects of exercise (Supplementary Fig. 12B, D). VPA-SDV-EX rats displayed unaltered mean intensity of NeuN in hippocampal and prefrontal regions compared with VPA. Conversely, VPA-SHAM-EX group showed significantly enhanced immunofluorescence intensity of NeuN in the DG and PFC, indicating that vagotomy may weaken the neuroprotective effects of exercise intervention.

## Discussion

Exercise has been well-documented as an effective intervention for ameliorating core symptoms of ASD, yet the underlying biological mechanisms remain incompletely elucidated. Our study systematically investigated the multi-dimensional effects of voluntary wheel running exercise on gut-brain axis regulation in ASD-like rodent models, encompassing gut microbiota composition, SCFAs metabolism, neuroactive substance homeostasis, and neuroinflammatory responses. We found that 6-week voluntary wheel running exercise effectively restores gut microbial architecture, normalizes SCFA production (particularly butyrate). These peripheral changes were associated with restored neuroactive substance balance in hippocampal and PFC regions, coupled with attenuated neuroinflammation, collectively contributing to behavioral improvements in ASD-like behaviors. To investigate the potential role of vagus-mediated gut-brain communication, we conducted complementary experiments using vagotomized ASD-like models. The surgical disruption of vagal afferents substantially attenuated exercise-induced therapeutic effects. Specifically, vagotomy blocked exercise’s capacity to suppress neuroinflammatory responses, correlating with attenuated behavioral recovery. Our results advance current understanding in two key dimensions: First, they support a novel mechanistic framework linking exercise-induced gut microbiota remodeling to neural circuit regulation through SCFAs-vagus-brain pathways. Second, intact vagal signaling serves as a key pathway for exercise-mediated gut-brain axis modulation in ASD pathophysiology, and the identification of vagal-associated mechanisms may offer valuable insights for developing targeted ASD interventions.

The present study employed prenatal VPA exposure in maternal rats to successfully establish offspring exhibiting core ASD-like phenotypes, including social interaction deficits and repetitive/stereotyped behaviors. Epidemiological evidence consistently demonstrates that prenatal VPA exposure elevates risks for neurodevelopmental impairments, congenital malformations, and ASD in offspring^9,44^. The underlying mechanisms are likely associated with perturbed neurodevelopment and sustained neuroinflammatory activation^45^. In this study, voluntary exercise was selected as the primary intervention due to its self-regulated nature, which aligns with the natural behavioral patterns of rodents. This approach helps avoid the anxiety-like behaviors and physiological stress responses commonly observed in forced exercise models. Mechanistically, voluntary exercise engages the dopaminergic reward circuitry, enhancing functional connectivity between prefrontal-limbic networks, and this neuroplastic adaptation may underlie its efficacy in ameliorating ASD-related social motivation deficits^46^. Andoh et al.^47^ demonstrated that voluntary exercise activates dentate granule cells and normalizes synaptic density in the hippocampal CA3 region, thereby improving social behavior, repetitive behaviors, and anxiety in ASD-like mice induced by maternal immune activation. Our behavioral assessment demonstrated that 6 weeks of voluntary wheel running exercise elicited multi-domain improvements in ASD-like phenotypes, including enhanced spatial learning/memory, reduced repetitive behaviors, and restored social preference. However, following vagotomy, the beneficial effects of voluntary wheel running on ASD-like behaviors were markedly attenuated, underscoring the critical role of vagal integrity in mediating the exercise-induced improvements.

Our findings corroborate previous studies that prenatal VPA exposure induces significant gut microbiota dysbiosis in offspring rats. This dysbiosis likely stems from VPA-triggered maternal immune activation, which promotes oxidative stress and sustained neuroinflammatory responses in offspring^48^. At the genus level, VPA-exposed rats exhibited decreased abundances of *Alloprevotella* and *Rodentibacter*, alongside increased *unidentified_Coriobacteriales*. Six weeks of voluntary wheel running altered the structure of gut microbiota and reversed genus-level dysbiosis. These microbial changes correlated with reduced repetitive behaviors and improved social abilities in ASD-like rats, aligning with established evidence that exercise remodels gut microbiota to enhance CNS function^49–51^. Previous studies have also confirmed that physical activity enhances microbial diversity^52–54^ and the synthesis of neuroprotective metabolites^55^. Our study revealed that vagotomy induces directional shifts in ASD-associated genera (such as *Negativibacillus*, *Bacteroides*, and *Parabacteroides*) while still attenuating the therapeutic effects of exercise. An interesting observation was that the genus *Romboutsia* consistently emerged as a key biomarker in the CONN group in the LEfSe analysis, whereas secondary discriminative taxa exhibited variability. This suggests that microbial characteristics may be context-dependent within multifactorial experimental designs.

SCFAs are crucial metabolites of the gut microbiota^56–58^, exerting regulatory effects on CNS function via the gut-brain axis^24^. While some enriched bacterial genera (e.g., *Bacteroides*, *Roseburia*) are known SCFA producers, other genera (particularly *Parasutterella* and *Alistipes*) may influence the gut-brain axis through alternative mechanisms. These mechanisms may include regulating the immune system, maintaining intestinal barrier integrity, or interacting with other bacteria in the microbial network to indirectly support a SCFA-favorable environment. Consistent with prior clinical studies^19,20,59^, this research identified significantly reduced levels of acetate, butyrate, caproate, and isocaproate in the feces and plasma of VPA-induced ASD-like rats. A 6-week voluntary wheel running exercise intervention restored systemic SCFA concentrations, with the most pronounced increase observed in butyrate levels. Butyrate has been shown to play a key role in maintaining microbial homeostasis, regulating SCFA synthesis/metabolism, and modulating diverse physiological processes^60,61^. Exercise-mediated SCFA levels in feces and plasma remain increased even after vagotomy; however, this restoration of SCFAs was not associated with behavioral improvements in the ASD-like model rats. As the primary pathway of gut-brain communication, the vagus nerve directly senses intestinal SCFAs changes via visceral afferents and transmits these signals to the CNS. Mechanistically, SCFAs activate FFARs on vagal afferent fibers, triggering neuronal impulses that project to the nucleus tractus solitarius (NTS) to modulate CNS function^24,62^. Previous studies indicated that subdiaphragmatic vagotomy abolishes butyrate’s effects on feeding behavior and brown adipose tissue metabolism^63^, while selective knockout of FFAR3 in vagal afferent blocks propionate-induced anorexia in mice^64^.

Studies in ASD animal models have implicated neuropathological abnormalities across multiple brain regions, including the hippocampus, amygdala, cerebral cortex, and PFC^65,66^. Cao et al.^67^, demonstrated reduced excitability of parvalbumin-expressing interneurons in the PFC of ASD-like mice, a perturbation potentially underlying social interaction deficit. Studies by Zhang et al.^68^, revealed that diminished excitatory synaptic transmission in pyramidal neurons of the PFC similarly recapitulates ASD-like behavioral phenotypes. These findings collectively suggest that dysregulated neuronal activity in PFC may constitute a critical neural substrate for ASD-associated social impairments. Previous evidence highlighted the hippocampus as a pivotal mediator of ASD core symptoms^69,70^, particularly its role in social behavior modulation^71,72^. Hippocampal neurogenesis deficits have been mechanistically linked to cognitive dysfunction and emotional dysregulation in ASD^73^. Rudie et al.^74^, observed that children with ASD exhibit reduced functional connectivity within the hippocampal network compared to neurotypical children, which may contribute to memory processing impairments and, in turn, exacerbate social communication deficits^75^. Recent studies indicated that impaired functional integration between the hippocampus and PFC may lead to abnormal information processing and behavioral output, exacerbating social and cognitive impairments in ASD^76^. Consistent with our rationale for focusing on these regions, our findings demonstrate that VPA-induced ASD-like rats exhibit significant neurobiological alterations in the prefrontal cortex and hippocampus.

Accumulating evidence implicates dysregulation of neurotransmitter systems and disrupted excitatory/inhibitory (E/I) balance as essential contributors to ASD pathophysiology^77–80^. Clinical and preclinical studies consistently link ASD-associated social behavioral deficits to altered neural circuit activity and neurotransmitter dynamics, particularly involving glutamate, gamma-aminobutyric acid (GABA), serotonin (5-HT), melatonin, and dopamine^81–87^. Perturbations in glutamatergic and GABAergic signaling may critically impair E/I equilibrium, potentially compromising information processing and leading to social behavioral deficits^9^. Studies in VPA-induced ASD models have revealed significantly reduced levels of neuroactive metabolites–including threonine, kynurenine, 5-hydroxyindoleacetic acid, and betaine aldehyde chloride in hippocampal and prefrontal tissues^88^. Our findings demonstrate that exercise intervention robustly modulates neuroactive substance profiles in these brain regions, with observed changes correlating strongly with amelioration of ASD-like behaviors. In contrast, the effects of vagotomy on exercise-induced neurochemical changes were subtle. Furthermore, we observed that exercise elicited divergent neuromodulatory patterns between the hippocampus and prefrontal cortex, which may be attributed to several factors. First, distinct regulatory mechanisms governing neuroactive substance synthesis/reuptake between the hippocampus and prefrontal cortex. Second, neural circuit architecture differences across brain regions result in differential neuroactive substance transmission and regulation pathways. Moreover, complex feedback mechanisms in neuroactive substance regulation mean that changes in one region can affect others via neural network feedback.

Neuroinflammatory activation has emerged as an independent risk factor in ASD pathogenesis and its associated comorbidities^89–92^. Convergent evidence from human neuropathological studies revealed aberrant activation of microglia and astrocyte across multiple brain regions in ASD patients^93–98^, a phenomenon recapitulated in our VPA-induced ASD model. Mirroring earlier findings where systemic propionate administration triggered microglial hyperactivation, neurotoxic cytokine release, and ASD-like behaviors in rodents^99^. Our study similarly exhibited pronounced microglial/astrocyte reactivity in hippocampal and prefrontal tissues, with microglia polarized toward a pro-inflammatory phenotype. Such overactivation of neuroinflammatory cells likely disrupts neuronal trophic support, destabilizes neuroactive substance homeostasis, and exacerbates synaptic dysfunction, collectively contributing to cognitive and social deficits. We have found that voluntary exercise intervention attenuated microglial reactivity and shifted their polarization toward an anti-inflammatory phenotype, concurrently reducing astrocyte reactivity. This exercise-mediated neuroimmune modulation may restore neurotrophic factor secretion and rebalance the neural microenvironment, thereby promoting neuroprotection and synaptic plasticity. However, vagotomy attenuated the anti-inflammatory effects of exercise and its behavioral benefits, suggesting that vagal signaling is a critical component of exercise-driven neuroimmune regulation. But how does a peripheral nerve signal influence brain-resident immune cells? The most direct route is the cholinergic anti-inflammatory pathway. VNS has been demonstrated to suppress microglial overactivation in neuroinflammatory conditions^30^, a process potentially mediated by the α7nAChR-dependent cholinergic anti-inflammatory pathways^33–35^. The communication between the gut microbiota and the brain, particularly via the microbiota-gut-brain axis, can be modulated by the vagus nerve to regulate inflammatory responses and has been implicated in a range of conditions, including neurodevelopmental, neuropsychiatric, and neurodegenerative disorders^36^. Intestinal enteroendocrine cells detect luminal chemical signals, such as alterations in SCFAs. These signals are relayed via enteric neurons to the vagus nerve, ultimately triggering anti-inflammatory responses^36^. Alterations in blood-brain barrier (BBB) permeability may permit peripheral immune signals to trigger neuroinflammation, a process that can be modulated by the vagus nerve. Evidence from both ASD patients and animal models indicates functional and structural impairments of the BBB^100–102^. When microglia within the brain parenchyma are exposed to damage-associated molecular patterns, they undergo phenotypic changes that lead to increased release of pro-inflammatory mediators, thereby exacerbating BBB dysfunction^103^. VNS has been demonstrated to upregulate α7nAChR, thereby inhibiting the release of pro-inflammatory cytokines and enhancing the integrity of the BBB^104^. Our data suggest that exercise-induced signals, potentially gut-derived SCFAs, likely rely on this intricate vagally-mediated circuitry to alleviate the neuroinflammatory state in the ASD brain.

Our findings provide a novel gut-brain axis perspective on the well-documented benefits of exercise. Voluntary physical activity is a potent non-pharmacological stimulator of vagal tone, as evidenced by increased heart rate variability in both humans and rodents^105,106^. This exercise-mediated boost in vagal function likely enhances the capacity for gut-to-brain communication. This is highly relevant to ASD pathophysiology, as individuals with ASD frequently exhibit reduced vagal tone, which correlates with the severity of social and sensory deficits^107^. Previous evidence implicated VNS in ameliorating ASD-related behaviors^27–29^, which may be related to the regulation of neuroinflammation by the vagal nerve. Our study positions voluntary exercise as a natural, accessible modality for “vagus nerve stimulation”. We propose that the efficacy of both exercise and VNS in ameliorating ASD symptoms may share a common final pathway: the suppression of maladaptive neuroinflammation via enhanced vagal signaling, ultimately leading to improved neuronal function and behavior.

Collectively, our study provides evidence that vagus nerve integrity is indispensable for exercise-mediated amelioration of ASD-like behaviors, offering novel insights into gut-brain axis signaling in ASD pathophysiology. These findings advance our understanding of neuromodulatory cross-talk between peripheral (microbial/SCFA) and central (neuroimmune) systems, with translational implications for developing multimodal ASD interventions targeting gut-brain communication. While these discoveries are compelling, several limitations warrant consideration. Firstly, while our subdiaphragmatic vagotomy procedure followed established protocols aimed at complete transection, we did not perform direct functional validation (e.g., cholecystokinin satiety test). Furthermore, the experimental design lacked sedentary vagotomized (VPA-SDV) and sedentary sham-operated (VPA-SHAM) control groups. Their inclusion would have allowed us to disentangle potential interactions between the VPA model itself and vagotomy, independent of exercise. Therefore, while the coherent blockade of exercise effects across behavioral and neuroinflammatory domains in VPA-SDV-EX rats provides circumstantial evidence for the necessity of abdominal vagal signaling, definitive proof of vagal mediation requires future studies that incorporate these additional controls and direct functional assays. Direct physiological validation of vagal afferent signaling, combined with selective chemogenetic or optogenetic manipulation of gut-innervating vagal neurons, will be crucial to precisely map the gut-brain circuits involved. Secondly, our reliance on the VPA-induced rodent model, though well-validated for ASD-like phenotypes, inherently simplifies the neurodevelopmental heterogeneity and clinical complexity of human ASD. Ethical and practical constraints preclude direct human experimentation, necessitating cautious extrapolation of findings until validated across complementary ASD models (e.g., SHANK3 knockout, maternal immune activation). Thirdly, while we identified critical interactions between SCFAs and vagal pathway in ASD, the precise molecular mechanism mediating SCFA–vagus–brain communication remains unresolved. Future investigations should delineate how gut-derived metabolites interface with vagal afferents to modulate neurochemical and neuroinflammatory cascades. Finally, the observed neuroactive substance alterations likely reflect dysregulation within specific neural circuits. Advanced techniques, such as in vivo calcium imaging or optogenetic interrogation, could map exercise-induced neuromodulation to prefrontal-hippocampal circuit dynamics, clarifying the pathophysiology contribute to ASD behavioral phenotypes.

**Fig. 13:**
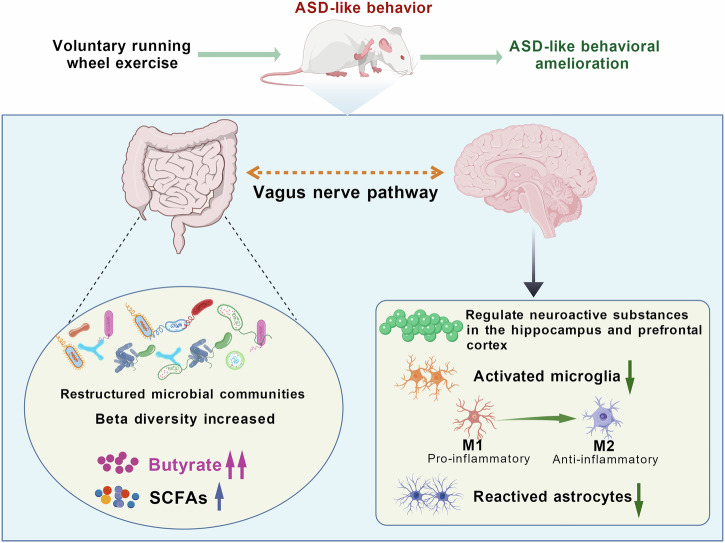
Schematic diagram illustrating how exercise-mediated improvement of ASD-like behaviors may require intact vagal signaling to coordinate gut-derived microbial and metabolic signals with central neuroadaptations (Figure created with BioGDP.com^117^).

Our findings demonstrated that 6 weeks of voluntary wheel running exercise ameliorates core behavioral deficits in ASD-like rats through multi-system adaptations encompassing gut microbiota restructuring, systemic SCFA elevation, and neuromodulatory/anti-inflammatory effects in the hippocampus and prefrontal cortex. Vagotomy attenuated exercise-induced restoration of neuroactive substance balance and neuroinflammatory resolution, substantially diminishing therapeutic efficacy (Fig. 13). These results highlight vagus nerve integrity as a key factor in exercise-driven gut-brain axis modulation, suggesting that peripheral microbial metabolic signals may require an intact vagal pathway to translate into central neuroadaptations.

## Methods

### Animals

Six-week-old specific pathogen free (SPF) Sprague-Dawley (SD) rats, comprising 36 males and 18 females, were procured from the Southern Medical University Laboratory Animal Center (Animal Use License No. SCXK(Yue) 2021-0041). Animals were housed in standardized conditions (ambient temperature 22 ± 1 °C, relative humidity 55 ± 5%, and a 12/12 h light/dark cycle), with ad libitum access to water and standard chow. Following a 2-week acclimatization period, females were paired with males overnight at a 1:2 ratio. Successful mating was confirmed by vaginal plug detection and sperm-positive vaginal smears the next morning, designated as embryonic day 1 (E1). Pregnant dams were subsequently single-housed. All procedures strictly adhered to the Guidelines for Welfare and Ethical Review of Laboratory Animals (GB/T 35892-2018) and the 3Rs principle (Replacement, Reduction, Refinement; Russell & Burch, 1959). Experimental protocols were approved by the Institutional Animal Care and Use Committee of Guangzhou Sport University (Approval No. 2022DWLL-11).

### VPA-induced ASD rat model

Fifteen pregnant SD rats received intraperitoneal injections of 600 mg/kg valproic acid sodium (VPA; Sigma-Aldrich P4543) at embryonic day 12.5 (E12.5), with male offspring designated as VPA-induced ASD models. Control cohorts comprised male progeny from three pregnant SD rats injected with an equal volume of saline under the same conditions. Following weaning, all animals were single-housed for the duration of the study to minimize the effects of social dominance on behavior and microbiota, and to allow for precise individual monitoring of voluntary wheel running activity in the exercise groups. At postnatal day 21, the male offspring of VPA-exposed dams were assessed for ASD-like behaviors using the Morris water maze, marble burying, open field, and three-chamber social tests, and those exhibiting typical behaviors were selected. The male offspring of saline-injected dams were used as the normal control group. To ensure robustness and consistency of behavioral phenotypes, VPA-exposed offspring were included in the study as the ASD-like rat models only if they met pre-defined selection criteria in these initial tests. The criteria required that an animal’s performance in specific core behavioral test (e.g., social interaction time in the three-chamber test, number of marbles buried) showed both a statistically significant difference from the CONN group (*P* < 0.05, Student’s t-test) and a substantial effect size, defined as a deviation greater than 1.5 standard deviations from the mean of the CONN group.

### Subdiaphragmatic vagotomy

Subdiaphragmatic vagotomy was performed as previously described^12,108,109^. Twenty-four ASD model rats at 4 weeks of age underwent subdiaphragmatic vagotomy (*n* = 12) or sham surgery (*n* = 12) under isoflurane anesthesia (5% induction, 1.5–2% maintenance via calibrated rodent anesthesia system). Following supine position and abdominal disinfection (75% alcohol), the skin and abdominal wall were incised at the mid-lateral aspect to gently expose the stomach and lower esophagus and to retract the intestine to gain access to the stomach. A ligature was placed around the esophagus at its entrance to the stomach to allow for gentle retraction and expose both vagal trunks. These were dissected, and all neural and connective tissue surrounding the esophagus below the diaphragm was removed to transect all small vagal branches. To ensure complete and permanent vagal transection and to prevent reinnervation, 2–4 mm segments of each vagal nerve branch were resected and removed. Sham-operated controls underwent identical procedures without actual vagus nerve transection to control for non-specific effects. Allow a 2-week postoperative recovery period to minimize surgical interference with subsequent experiments, followed by 6 weeks of voluntary wheel running exercise intervention.

### Voluntary wheel running exercise

From 6 weeks of age, rats subjected to exercise intervention were housed in cages equipped with voluntary running wheels (dimensions: 42 × 26 × 19 cm; wheel diameter 36 cm, running surface width 10 cm). Following a 3-day acclimatization period to habituate to wheel access, a 6-week voluntary wheel running exercise was initiated. An automated recording system with sensors (XR-PL107, Shanghai Xinruan Information Technology Co., Ltd., China) continuously monitored daily running activity, logging total distance (km) and duration (min).

### Experimental groups

In this study, male offspring were assigned to the following experimental groups: CONN group (offspring from saline-injected dams, sedentary control), VPA group (offspring from VPA-injected dams, sedentary), VPA-EX group (offspring from VPA-injected dams, subjected to 6 weeks of voluntary wheel running exercise), VPA-SDV-EX group (offspring from VPA-injected dams, underwent subdiaphragmatic vagotomy followed by exercise), VPA-SHAM-EX group (offspring from VPA-injected dams, underwent sham surgery followed by exercise).

### Behavioral assessment

All rats underwent pre- and post-intervention behavioral assessment in dedicated, sound-attenuated behavioral testing suites maintained under strictly controlled ambient conditions. The tests were conducted in the following order to minimize stress and interference: On postnatal day 21 (P21), each rat was first subjected to the open-field test (OFT), and immediately following the OFT session in the same arena, spontaneous self-grooming behavior was video-recorded for 10 min for subsequent analysis. On P22, the marble burying test (MBT) was performed. From P23 to P27, a daily split-session protocol was employed: the three-chamber social test (TST) was administered in the morning, followed by the Morris water maze (MWM) test in the afternoon, with a 3-hour interval between the two tests. All experimental data were analyzed using Visu Track Behavioral Analysis System (Shanghai Xinruan Information Technology Co., Ltd., China). All tests were conducted by two skilled investigators who blinded to the experimental groups. To ensure the integrity of the behavioral data, pre-defined exclusion criteria were applied prior to statistical analysis. For the open-field and three-chamber social tests, any animal that exhibited a profound freeze response (operationally defined as a total distance traveled of less than 1 m in the test) was excluded from the analysis for that specific test. This criterion was applied objectively and consistently across all experimental groups by an experimenter blinded to treatment conditions.

#### Morris water maze test

Spatial navigation and learning memory were assessed using the Morris water maze. The apparatus consisted of a circular pool (120 cm in diameter) filled with water maintained at 25 ± 1 °C. A hidden escape platform (10 cm in diameter) was submerged 1 cm below the water surface in the southeast quadrant, with spatial cues positioned around the room. The testing protocol comprised two phases: place navigation and spatial probe testing. Rats underwent a 120 s pretraining session 24 h prior to formal testing, during which they were guided to the platform and allowed to remain for 15 s. The place navigation test was then conducted over four consecutive days. Each day, rats performed four trials starting from four different quadrants, with an escape latency cutoff of 120 s. Animals that failed to locate the platform within this time were guided to it and remained there for 30 s. After the place navigation test, a spatial probe test was performed by removing the platform and releasing each rat from a novel quadrant. During the 120-s probe test, the following parameters were recorded: the time spent in the target quadrant, the distance swum in the target quadrant, the number of crossings over the original platform location, and the swimming speed. All behavioral analyses were conducted by investigators blinded to experimental groups.

#### Self-grooming test

Spontaneous self-grooming behavior, a core repetitive behavior in ASD models, was quantified under controlled conditions. Each rat was individually placed in a clean, empty standard cage (100 × 100 × 40 cm) without bedding. The behavior of rats was video-recorded for 10 min for subsequent analysis. Grooming bouts were defined as starting when the rat began licking or scratching its body with its forepaws and ending when all paws returned to the cage floor or the animal initiated a different behavior (e.g., sniffing or rearing). The total duration and frequency of grooming bouts were scored manually by an experimenter blinded to treatment assignments. Between trials, cages were thoroughly cleaned with 75% ethanol to eliminate olfactory cues.

#### Marble burying test

Repetitive/stereotyped behaviors were evaluated using the marble burying test. Clean cages (42 × 26 × 19 cm) were filled with 5 cm depth of fresh corncob bedding, upon which 20 glass marbles were arranged in a consistent 4 × 5 grid pattern. Each rat was placed individually into the testing cage for a 30-min session. Following the test, marbles were considered buried if at least two-thirds of their volume was covered by bedding. The number of buried marbles was quantified by two independent observers blinded to the experimental groups. Marbles were sanitized between trials with 70% ethanol to eliminate olfactory cues.

#### Open-field test

Spontaneous locomotor activity and exploratory behavior were assessed using the open-field test. The test was conducted in a square arena (100 × 100 × 40 cm) under dim lighting conditions (50 lux). Each rat was placed in the center of the arena and allowed to explore freely for 10 min. Locomotor activity was recorded and analyzed using Visu Track behavioral analysis software (Shanghai Xinruan Information Technology Co., Ltd., China). The parameters measured included total distance traveled, average speed, and time spent in the central zone. Between trials, the arena was thoroughly cleaned with 75% ethanol to eliminate olfactory cues.

#### Three-chambered social test

Sociability and social novelty preference were assessed using a three-chamber apparatus (150 cm × 50 cm × 40 cm). The arena was divided into three compartments by transparent partitions with central openings to allow free exploration. Cylindrical wire cages (height: 20 cm; diameter: 10 cm; bar spacing: 1 cm) were positioned in the left and right chambers to contain stimulus animals. The testing protocol consisted of three phases: (1) Habituation: the test rat explored the empty apparatus for 5 min; (2) Sociability test: an unfamiliar conspecific (Stranger 1) was enclosed in the left wire cage while the right remained empty, with 10 min of exploration recorded; (3) Social novelty test: a second novel mouse (Stranger 2) was introduced to the previously empty cage, with another 10 min of recording. Social interaction was quantified as time spent sniffing each wire cage using Visu Track behavioral analysis software (Shanghai Xinruan Information Technology Co., Ltd., China). The preference index of each animal is defined as (Time Stranger 1 - Time metal cage)/(Time Stranger 1 + Time metal cage) for the sociability test, and (Time Stranger 2 - Time Stranger 1)/(Time Stranger 2 + Time Stranger 1) for the social novelty test. The apparatus and cages were thoroughly cleaned with 75% ethanol between trials to eliminate olfactory cues.

### Sample collection

#### Fecal samples

Fresh fecal pellets were collected via anal swab using sterile cryovials immediately post-exercise intervention, flash-frozen in liquid nitrogen for 5–10 min, and stored at −80 °C for subsequent 16S rRNA gene sequencing analyses and SCFA profiling.

#### Plasma samples

Following a 12 h fasting period, rats were anesthetized with 1% sodium pentobarbital (1 mL/100 g, i.p.). Blood was drawn from the abdominal aorta into EDTA-coated vacutainers, inverted gently for anticoagulation, and centrifuged at 3000 × g for 10 min at 4 °C. Plasma aliquots were stored at −80 °C for SCFA profiling.

#### Hippocampus and prefrontal cortex tissues

Eight randomly selected rats per group were decapitated after euthanasia and blood collection. Brains were rapidly extracted, rinsed in ice-cold saline, and dissected on a pre-chilled platform. The hippocampus and prefrontal cortex were carefully dissected out, snap-frozen in liquid nitrogen, and stored at -80 °C for HPLC-MS neuroactive substance quantification.

#### Immunofluorescence tissue preparation

Four rats per group were deeply anesthetized with 1% sodium pentobarbital (1 mL/100 g, i.p.) and perfused transcardially with cold phosphate-buffered saline (PBS) followed by 4% paraformaldehyde (PFA). The brain samples were post-fixed with 4% PFA (24 h, 4 °C) and subsequently immersed in 20 and 30% sucrose in PBS for 24 h, respectively. Coronal sections of the hippocampus and prefrontal cortex (40 μm) were cut with a cryostat (CM1950; Leica, Germany) at −20°C, and stored in antifreeze solution (−20 °C) for future immunofluorescence analysis.

### 16S rRNA gene sequencing analyses

DNA from each sample was extracted, the V3-V4 (341F-806R) primer region of 16S rDNA was selected for amplification, and the library was double-ended sequencing using the Illumina NovaSeq 6000 platform (Illumina Inc., San Diego, CA, USA). The Sequencing and bioinformatics analyses were performed by Metware Biotechnology Co., Ltd., Wuhan, China.

#### DNA extraction and purification from fecal samples

Fecal genomic DNA was extracted and purified using the MagPure Soil DNA LQ Kit (D6356-03, Magen Biotechnology, Guangzhou, China) following the manufacturer’s protocol. The purified DNA was eluted in an appropriate elution buffer and subjected to quality control assessments. DNA integrity was evaluated through 1.2% agarose gel electrophoresis under standard conditions. Quantitative analysis was performed using a Nanodrop spectrophotometer (ND-2000, Thermo Fisher Scientific, Waltham, MA, USA) to determine DNA concentration and purity.

#### Amplicon sequencing

The V3-V4 hypervariable regions of bacterial 16S rRNA gene were amplified using specific primers 341 F (5′-CCTAYGGGRBGCASCAG-3′) and 806 R (5′-GGACTACNNGGGTATCTAAT-3′) under rigorously controlled thermocycling conditions. Amplified products were purified using VAHTS DNA Clean Beads (Vazyme Biotech, Nanjing, China) to remove primer dimers and non-target fragments. Purified amplicons were quantified fluorometrically using the Quant-iT PicoGreen dsDNA Assay Kit (Thermo Fisher Scientific, Waltham, MA, USA). Final libraries were pooled in equimolar ratios based on sequencing depth requirements and subjected to paired-end sequencing (2 × 300 bp) on an Illumina NovaSeq PE250 platform (Illumina Inc., San Diego, CA, USA). Raw sequencing reads were processed through quality control using fastp (v0.22.0, https://github.com/OpenGene/fastp) to obtain high-quality reads by removing low-quality sequences (quality score < 20), adapters, and reads shorter than 150 bp. High-quality paired-end reads were then merged into contiguous sequences (Clean Tags) using FLASH (v1.2.11; http://ccb.jhu.edu/software/FLASH/). Chimeric sequences were detected and removed by reference-based alignment against the SILVA 138.1 16S rRNA gene database (https://www.arb-silva.de) using vsearch (v2.22.1; https://github.com/torognes/vsearch/), resulting in high-confidence Effective Tags for subsequent analyses.

#### Sequence data analysis

Amplicon Sequence Variants (ASVs) were generated through denoising pipelines implemented in QIIME 2 (v2023.2, https://qiime2.org) using DADA2 (v1.26.0) and Deblur (v1.1.1) plugins with default parameters. Taxonomic classification was performed using Mothur (v1.48; University of Michigan, Ann Arbor, MI, USA) against the SILVA SSUrRNA reference database (v138.1, https://www.arb-silva.de) with confidence thresholds of 0.8–1.0. The top 10 most abundant taxa at each taxonomic rank (Phylum to Species) were visualized as stacked bar plots using ggplot2 (v3.4.2) in R (v4.2.0; R Foundation, Vienna, Austria). Alpha diversity metrics (Observed_ASVs, Chao1, Shannon, Simpson, ACE, Goods-coverage, PD_whole_tree) were calculated using phyloseq (v1.40.0) and vegan (v2.6.2) packages. Sequencing depth adequacy was evaluated via rarefaction curves, while species richness and evenness were assessed through Rank abundance curves. The β-diversity distance measurements, including Bray-Curtis, unweighted UniFrac, and weighted UniFrac, were performed to determine the structural variation in microbial communities across samples and then visualized by principal coordinate analysis (PCoA). To identify inter-group differences in microbial community structure, taxonomic profiles were subjected to Linear Discriminant Analysis Effect Size (LEfSe). Briefly, LEfSe uses the non-parametric Kruskal–Wallis test (FDR-adjusted *P* < 0.05) to identify features with significant abundance differences among classes, followed by pairwise Wilcoxon tests to assess whether these differences are consistent across subclasses. Finally, Linear Discriminant Analysis (LDA) is used to estimate the effect size of each differentially abundant feature. Taxa achieving LDA scores >2 with FDR-adjusted *P* < 0.05 were designated as group-specific biomarkers, visualized through cladograms and LDA bar plots generated in MicrobiomeAnalyst (v4.1). Statistical validation incorporated Welch’s t-test (phyloseq v1.40.0) for taxon-level comparisons and Analysis of Similarities (ANOSIM; vegan v2.6.4) with 9999 permutations based on Bray-Curtis distances. The functional potential of the gut microbial communities was predicted from the 16S rRNA gene amplicon data using PICRUSt2 (Phylogenetic Investigation of Communities by Reconstruction of Unobserved States) software (version 2.5.0). The resulting predicted metagenome data, represented as the abundance of KEGG Orthologs (KOs), was utilized in the correlation analysis with short-chain fatty acid levels.

### SCFAs levels analysis

A total of nine SCFAs, including acetic acid, propionic acid, isobutyric acid, butyric acid, isovaleric acid, valeric acid, 2-methylvaleric acid, isocaproic acid, and caproic acid, in fecal and plasma samples were detected by MetWare (http://www.metware.cn/) based on the Agilent 7890B-7000D GC-MS/MS platform^110–112^.

#### Sample preparation and extraction

Twenty milligrams of fecal samples were accurately weighed and placed in a 2 mL EP tube. 1 mL of phosphoric acid (0.5% v/v) solution and a small steel ball were added to the EP tube. The samples were ground uniformly, then vortexed for 10 min and ultrasonicated for 5 min. 100 μL of supernatant was moved into 1.5 mL centrifugal tube after the mixture was centrifuged with a speed of 12,000 r/min for 10 min at 4 °C. 500 μL of MTBE (containing internal standard) solution was added to the centrifugal tube, and the mixture was vortexed for 3 min followed by ultrasonicating for 5 min. After that, the mixture was centrifuged at a speed of 12,000 r/min for 10 min at 4 °C. The supernatant was collected and used for GC-MS/MS analysis.

#### GC-MS/MS analysis

Agilent 7890B gas chromatograph coupled to a 7000D mass spectrometer with a DB-FFAP column (30 m length × 0.25 mm i.d. × 0.25 μm film thickness, J&W Scientific, USA) was employed for GC-MS/MS analysis of SCFAs. Helium was used as carrier gas, at a flow rate of 1.2 mL/min. Injection was made in the split mode with a split ratio of 5:1, and the injection volume was 1 μL. The oven temperature was held at 50 °C for 1 min, raised to 220 °C at a rate of 18 °C/min, and held for 5 min. All samples were analyzed in multiple reaction monitoring mode. The injector inlet and transfer line temperature were 250 °C and 230 °C, respectively. The mass spectrum peak area of the sample analyte was substituted into the linear equation to calculate the concentration.

For plasma samples, the samples were thawed and vortexed for 1 min before analysis. Fifty microliters of the samples were added to a 1.5 mL EP tube, and 100 μL of phosphoric acid (0.5%, v/v) solution was added to the EP tube. The mixture was vortexed for 3 min. 150 μL MTBE (containing internal standard) solution was added. The mixture was vortexed for 3 min and ultrasonicated for 5 min. After that, the mixture was centrifuged at a speed of 12,000 r/min for 10 min at 4 °C. The other steps are the same as above.

### Analysis of neuroactive substances

Neuroactive substances in the hippocampus and prefrontal cortex were detected by MetWare (http://www.metware.cn/) based on the AB Sciex QTRAP 6500 LC-MS/MS platform^113–116^.

#### Sample preparation and extraction

After the sample was thawed and smashed, an amount of 0.05 g of the sample was mixed with 500 µL of 70% methanol/water. The sample was vortexed for 3 min under the condition of 2500 r/min and centrifuged at 12,000 r/min for 10 min at 4 °C. Take 300 μL of supernatant into a new centrifuge tube and place the supernatant in −20 °C refrigerator for 30 min. Then the supernatant was centrifuged again at 12,000 r/min for 10 min at 4 °C. After centrifugation, transfer 200 μL of supernatant for further LC-MS analysis.

#### UPLC Conditions

The sample extracts were analyzed using an LC-ESI-MS/MS system (UPLC, ExionLC AD, https://sciex.com.cn/; MS, QTRAP® 6500+ System, https://sciex.com/). The analytical conditions were as follows, HPLC: column, Waters ACQUITY UPLC HSS T3 C18 (100 mm × 2.1 mm i.d., 1.8 µm); solvent system, water with 0.1% formic acid (A), acetonitrile with 0.1% formic acid (B); The gradient was started at 5% B (0 min), increased to 95% B (0–8 min), 95% B (8–9.5 min), finally ramped back to 5% B (9.6–12 min); flow rate, 0.35 mL/min; temperature, 40 °C; injection volume: 2 μL.

#### ESI-MS/MS Conditions

AB 6500 + QTRAP® LC-MS/MS System, equipped with an ESI Turbo Ion-Spray interface, operating in both positive and negative ion modes and controlled by Analyst 1.6 software (AB Sciex). The ESI source operation parameters were as follows: ion source, turbo spray; source temperature 550 °C; ion spray voltage (IS) 5500 V (Positive), −4500 V (Negative); curtain gas (CUR) was set at 35.0 psi; DP and CE for individual MRM transitions was done with further DP and CE optimization. A specific set of MRM transitions was monitored for each period according to the neuroactive substances eluted within this period.

### Immunofluorescence (IF) staining

Coronal sections containing the hippocampal formation and prefrontal cortex (PFC) were selected based on the rat brain atlas of Paxinos and Watson (2014). The hippocampus was selected between approximately −3.60 and −3.96 mm posterior to bregma. Within this region, specific subregions (CA1, CA2, CA3, DG) were identified and analyzed. The prefrontal cortex was selected between approximately +3.00 and +3.72 mm anterior to bregma. Sections within these ranges were collected for all subsequent immunofluorescence analyses to ensure consistency across animals. Immunofluorescence staining was performed on free-floating sections. The sections of the hippocampus and prefrontal cortex were rinsed three times (15 min each) with 0.1 M PBS. Sections were then permeabilized and blocked for 1.5 h at room temperature in 0.1 M PBS with 0.3% Triton X-100 and 5% BSA. Following blocking, sections were incubated with primary antibodies in 0.1 M PBS with 0.3% Triton X-100 and 5% BSA at 4 °C overnight. For negative controls, the primary antibodies were omitted and replaced with the same amount of normal serum from the same species. The sections were rinsed three times with 0.1 M PBS and then incubated with secondary antibodies in 0.1 M PBS with 0.3% Triton X-100 and 5% BSA for 2 h at room temperature under light-protected conditions. Following secondary antibody removal and three additional PBS rinses, nuclear counterstaining was performed using 4′,6-diamidino-2-phenylindole (DAPI) for 10 min at room temperature. Finally, sections were mounted on poly-L-lysine-coated slides and cover-slipped.

The primary antibodies used in this study were as follows: rabbit anti-Iba1 antibody (1:1000, 019-19741, Wako), mouse anti-iNOS antibody (1:100, sc-77271, Santa Cruz), mouse anti-Arginase-1 monoclonal antibody (1:200, 66129-1-Ig, Proteintech), mouse anti-GFAP (2E1) antibody (1:200, sc-33673, Santa Cruz), mouse anti-NeuN antibody (1:1000, ab104224, Abcam). The secondary antibody conjugated with the fluorochrome included Goat Anti-Rabbit IgG (H + L) Secondary Antibody, DyLight™ 488 (1:2000, 35552, Invitrogen), and Goat Anti-Mouse IgG (H + L) Alexa Fluor Plus555 (1:400, A21422, Thermo Fisher).

The immunostaining was imaged using the THUNDER imaging system (DMi8, Leica, Germany) with LAS X software (Leica). Negative controls omitting primary antibodies confirmed staining specificity. Mean fluorescence intensity and number of positive fluorescent cells in hippocampal and prefrontal cortex regions were measured in Image J software by selecting regions of interest using the ROI manager.

### Statistical analysis

Data processing, statistical analysis, and visualization were performed using GraphPad Prism 9.0 (GraphPad Software, San Diego, CA, USA) and Microsoft Excel (Microsoft Corp., Redmond, WA, USA). Normality and homogeneity of variance were first assessed via Shapiro-Wilk and Levene’s tests, respectively. Datasets fulfilling both parametric assumptions (normally distributed with equal variances) were analyzed by one-way ANOVA with least significant difference (LSD) post hoc comparisons. Non-normally distributed data underwent logarithmic or square root transformations followed by reassessment of parametric assumptions; transformed data meeting criteria were subjected to parametric analysis, while persistent violations triggered nonparametric Kruskal-Wallis testing. Continuous variables are presented as mean ± standard error of the mean (SEM), with statistical significance defined as *P* < 0.05.

Spearman correlation analysis was performed to assess potential associations between behavioral indices and differential gut microbiota, SCFAs levels, and neuroactive substance levels. The same method was applied to evaluate correlations between altered gut microbiota and SCFA profiles. Relationships between behavioral indices and neuroinflammatory markers were analyzed using the Mantel test. Correlation networks were constructed to visualize interactions among SCFAs, neuroactive substances, and neuroinflammatory markers. All correlation cluster heatmaps and network diagrams were implemented through the Metware Cloud Platform (Metware Biotechnology Co., Ltd., Wuhan, China).

## Supplementary information

Supplementary Information

## Data Availability

The 16S RNA gene sequencing data reported in this study have been deposited in the Genome Sequence Archive (GSA) in National Genomics Data Center (NGDC), China National Center for Bioinformation / Beijing Institute of Genomics, Chinese Academy of Sciences. The data are accessible via the GSA database (https://bigd.big.ac.cn/gsa) under accession number CRA025042.
