# Supplementary material for: Voluntary wheel running exercise attenuates VPA-induced ASD-like behaviors in male rats: implication of the vagal pathway of the gut-brain axis
Source: NPJ Biofilms Microbiomes. 2026 Mar 20;12:94. doi: 10.1038/s41522-026-00962-4 (PMC13168623; doi:10.1038/s41522-026-00962-4)
Supplement: Supplementary file 1 — Supplementary Information [file 41522_2026_962_MOESM1_ESM.pdf]

Voluntary Wheel Running Exercise Attenuates VPA-Induced ASD-like Behaviors in Male Rats: Implication of the Vagal Pathway of the Gut-Brain Axis

Yinhua Li[1, 2, 3], Jiugen Zhong[1, 3, 6], Yingying Shen[1], Jiaheng Gong[1], Yanqing Feng[1], Wanting Lan[1], Xiaohui Hou[1, 3, 4, 5, *]

Supplementary Figures

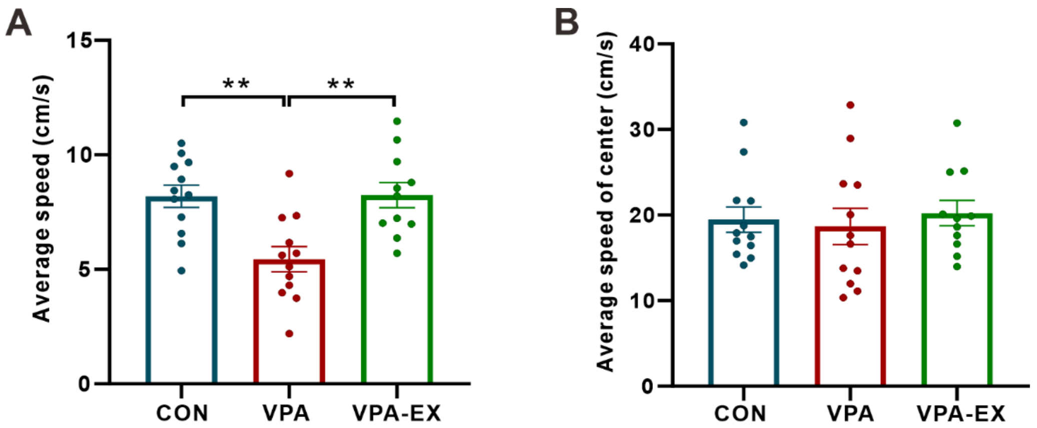

Supplementary Figure 1. The average speed **(A)**, and average speed in the center **(B)** in the open-field test. * $P < 0.05$, ** $P < 0.01$, *** $P < 0.001$, **** $P < 0.0001$.

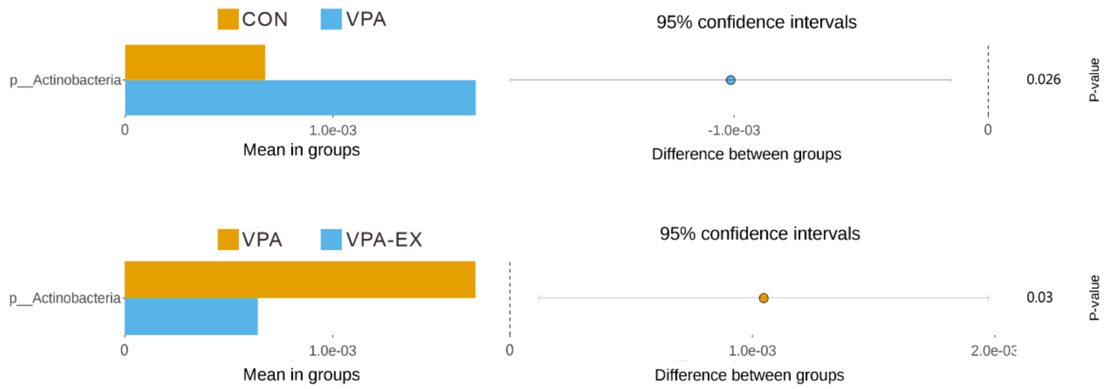

Supplementary Figure 2. Compositional differences at phylum level in the gut microbiota across CON, VPA and VPA-EX groups.

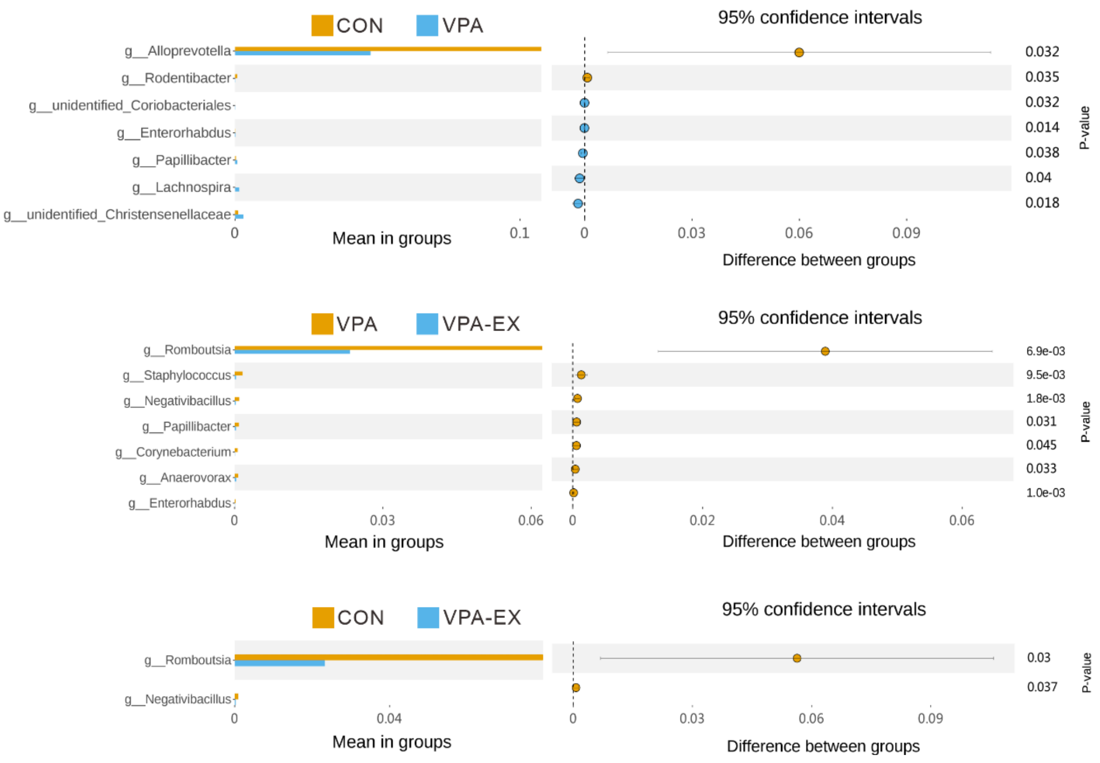

Supplementary Figure 3. Compositional differences at genus level in the gut microbiota across CON, VPA and VPA-EX groups.

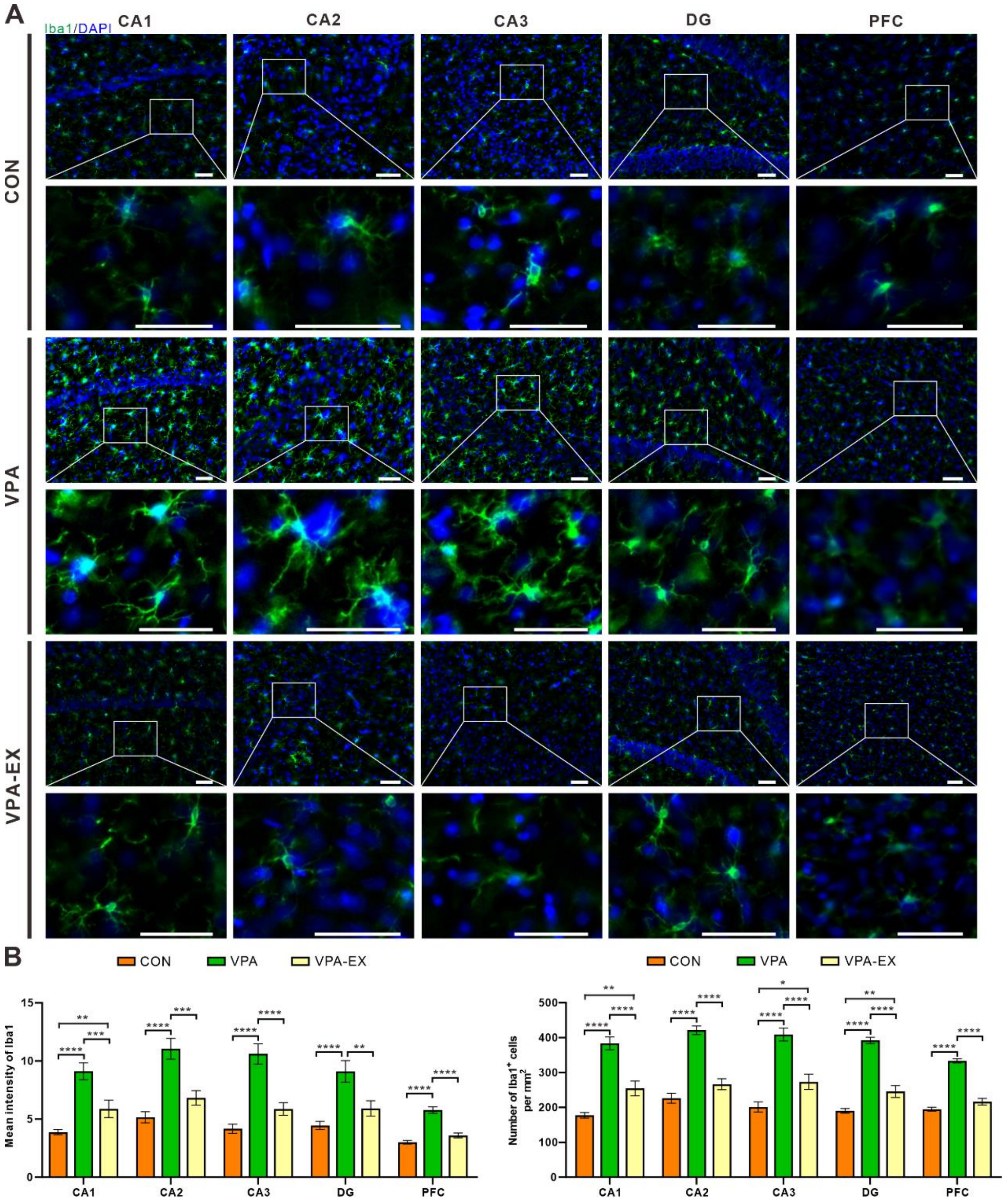

Supplementary Figure 4. Exercise affected microglia reactivity in the hippocampus and prefrontal cortex of VPA-induced ASD-like rats. (**A**) Immunofluorescence for Iba1 (green) with DAPI (blue) nuclear counterstain in the CA1, CA2, CA3 subregions and dentate gyrus (DG) of the hippocampus as well as in the prefrontal cortex (PFC). (**B**) Mean intensity and number of Iba1^{+} cells per mm^2. Data were expressed as mean ± SEM. Scale bar = 100 μm, * $P < 0.05$, ** $P < 0.01$, *** $P < 0.001$ and **** $P < 0.0001$. All experiments were performed in triplicate, four animals from each group and

twelve randomly selected images.

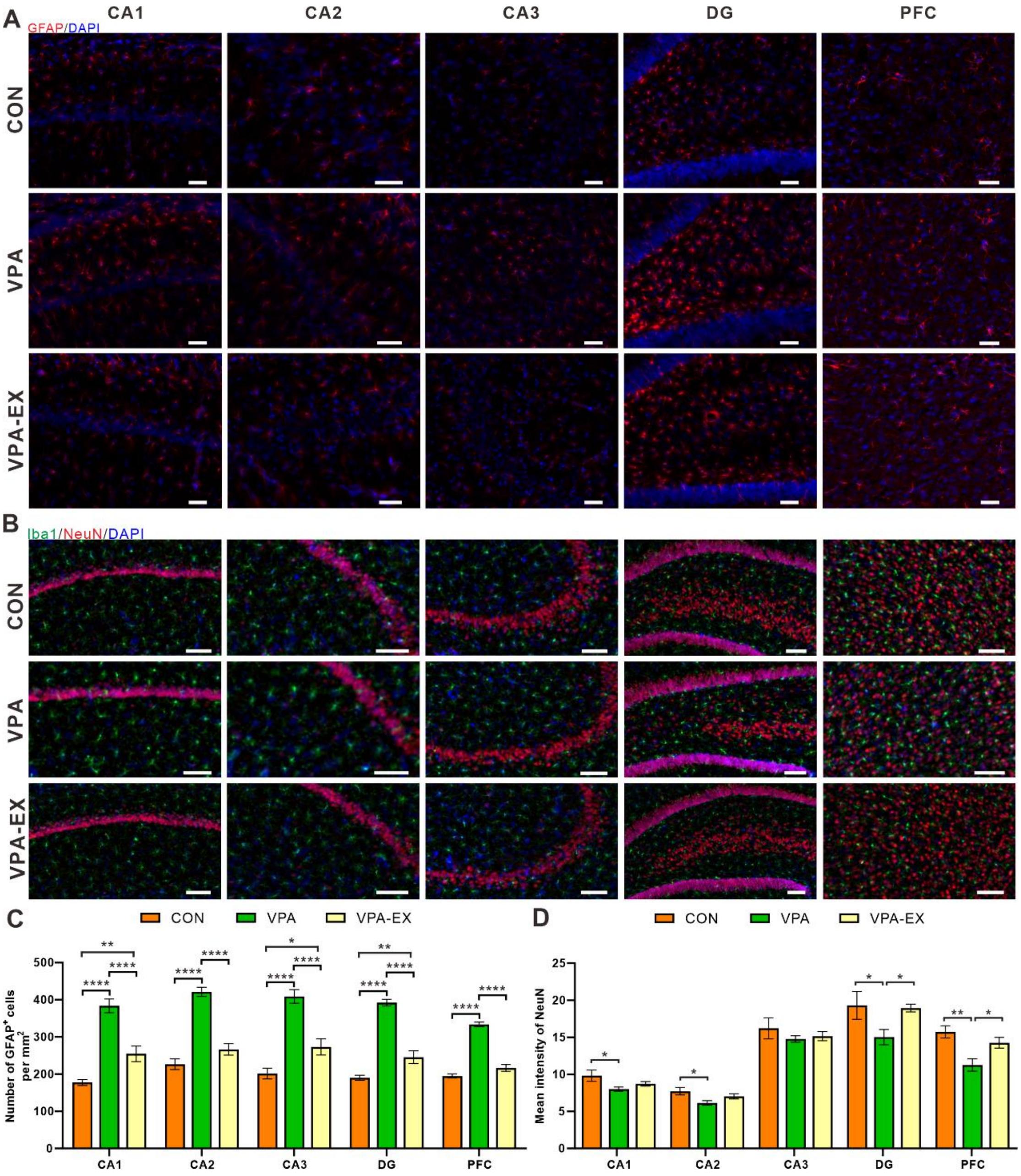

Supplementary Figure 5. Exercise affected astrocyte reactivity and neuronal expression in the hippocampus and PFC of VPA-induced ASD-like rats. **(A)** Immunofluorescence for GFAP (red) with DAPI (blue) nuclear counterstain in the CA1, CA2, CA3 subregions and DG of the hippocampus as well as in the PFC. **(B)** Immunofluorescence for NeuN (red) with DAPI (blue) nuclear counterstain in the CA1, CA2, CA3 subregions and DG of the hippocampus as well as in the PFC. **(C)** Number of $GFAP^+$ cells per mm^2. **(D)** Mean intensity of $NeuN^+$ cells per mm^2. Data

were expressed as mean ± SEM. Scale bar = 100 μm, $^{*}P < 0.05$, $^{**}P < 0.01$, $^{***}P < 0.001$ and $^{****}P < 0.0001$. All experiments were performed in triplicate, four animals from each group and twelve randomly selected images.

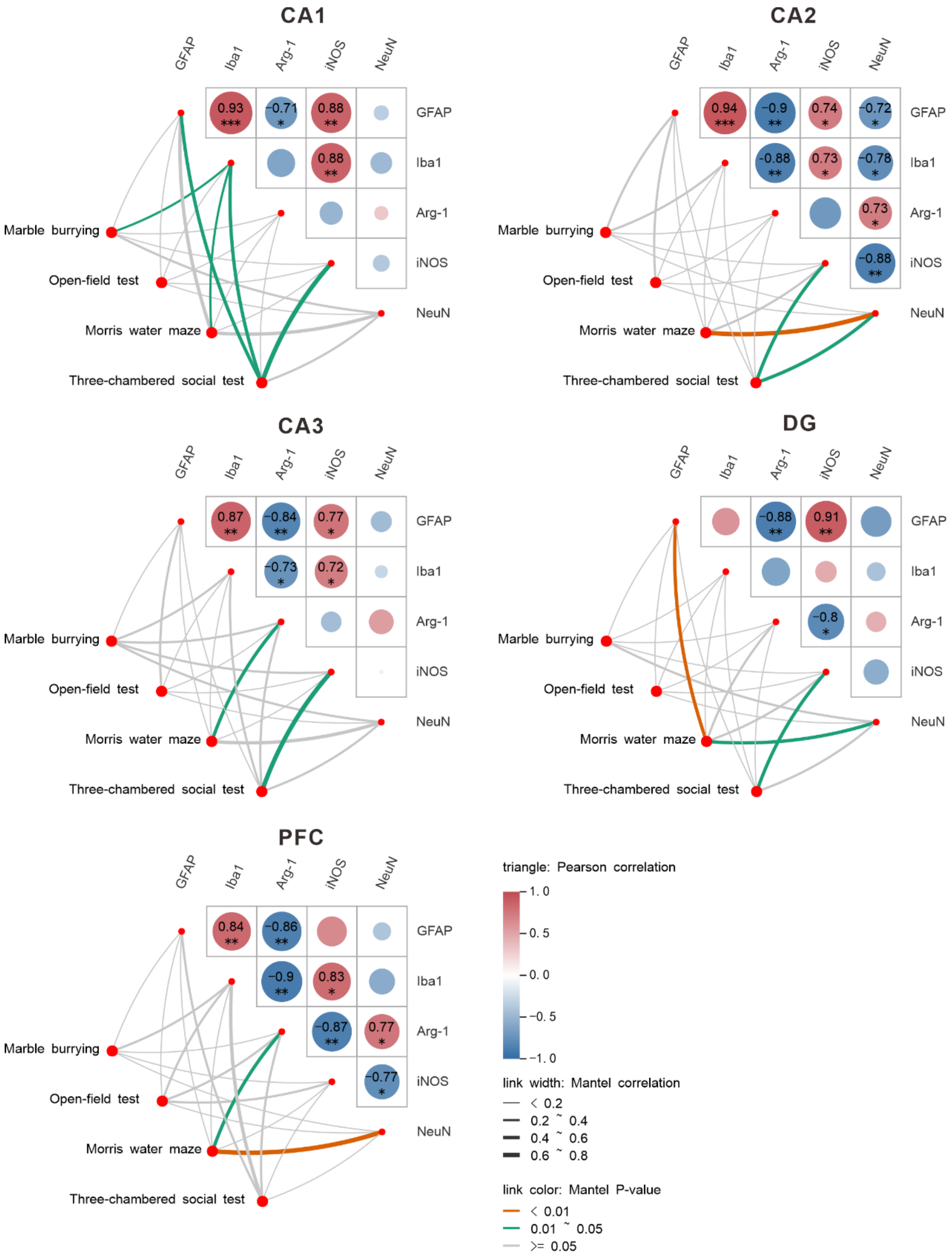

Supplementary Figure 6. The Mantel test analysis of the correlation between ASD-like behaviors and neuroinflammation. The color intensity in the heatmap represents the Pearson correlation coefficients between neuroinflammatory factors. Red represents positive correlation, and blue represents negative correlation, the intensity of the color indicates the degree of correlation. The size of the circles in the heatmap corresponds to the significance levels of the neuroinflammatory factors, with larger circles denoting higher significance. In the network diagram, the thickness of the connections is directly proportional to the correlation strength as determined by the Mantel test, thicker lines indicate a stronger correlation. The color of the lines reflects the significance of the Mantel test, with red indicating a higher absolute value of the correlation coefficient and a smaller corresponding P-value, suggesting a stronger association between neuroinflammation and ASD-like behaviors.

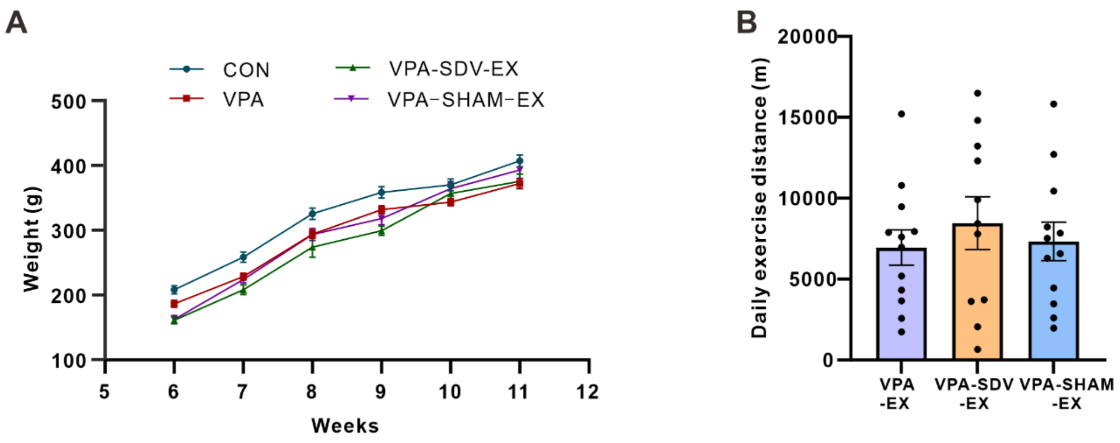

Supplementary Figure 7. **(A)** Body weight trajectory of rats over a 6-week period. **(B)** Daily running distance recorded during the voluntary wheel running exercise intervention. Data are presented as mean ± SEM.

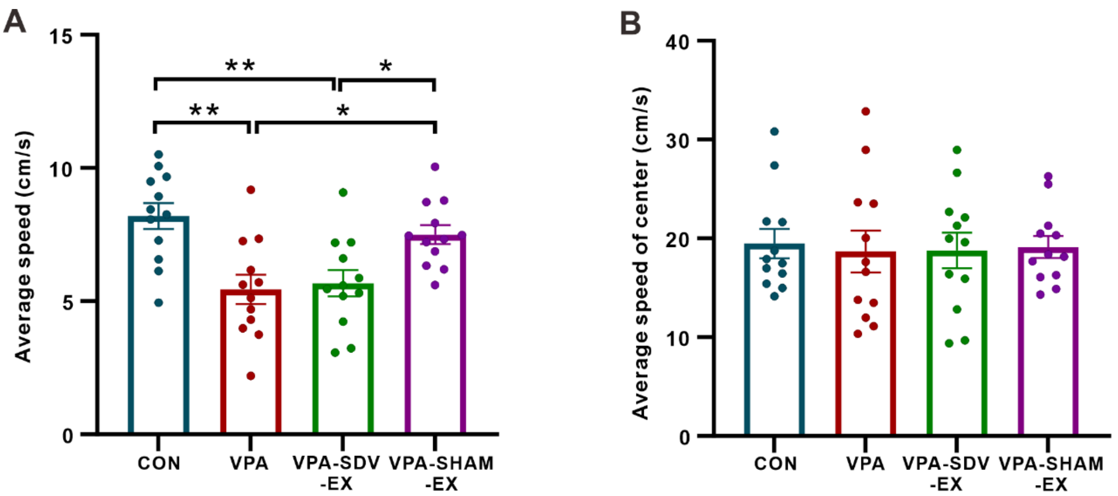

Supplementary Figure 8. The average speed **(A)**, and average speed in the center **(B)** in the open-field test. * $P < 0.05$, ** $P < 0.01$, *** $P < 0.001$, **** $P < 0.0001$.

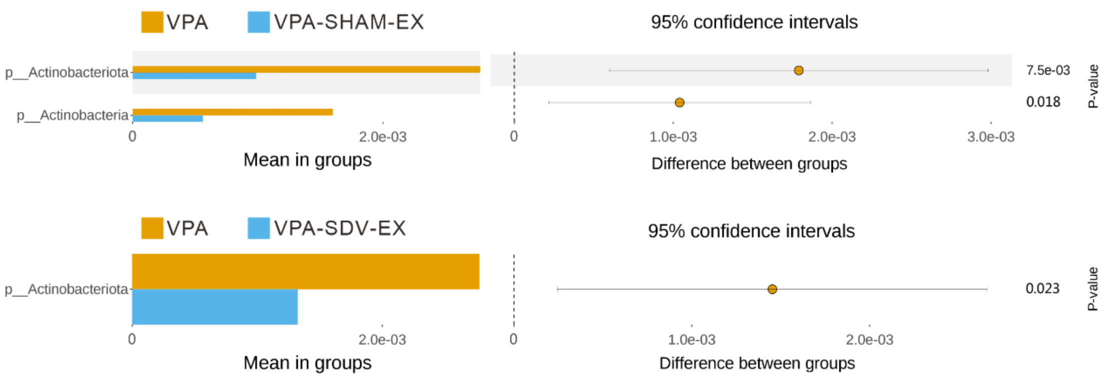

Supplementary Figure 9. Compositional differences at phylum level in the gut microbiota across CON, VPA, VPA-VGX-EX and VPA-SHAM-EX groups.

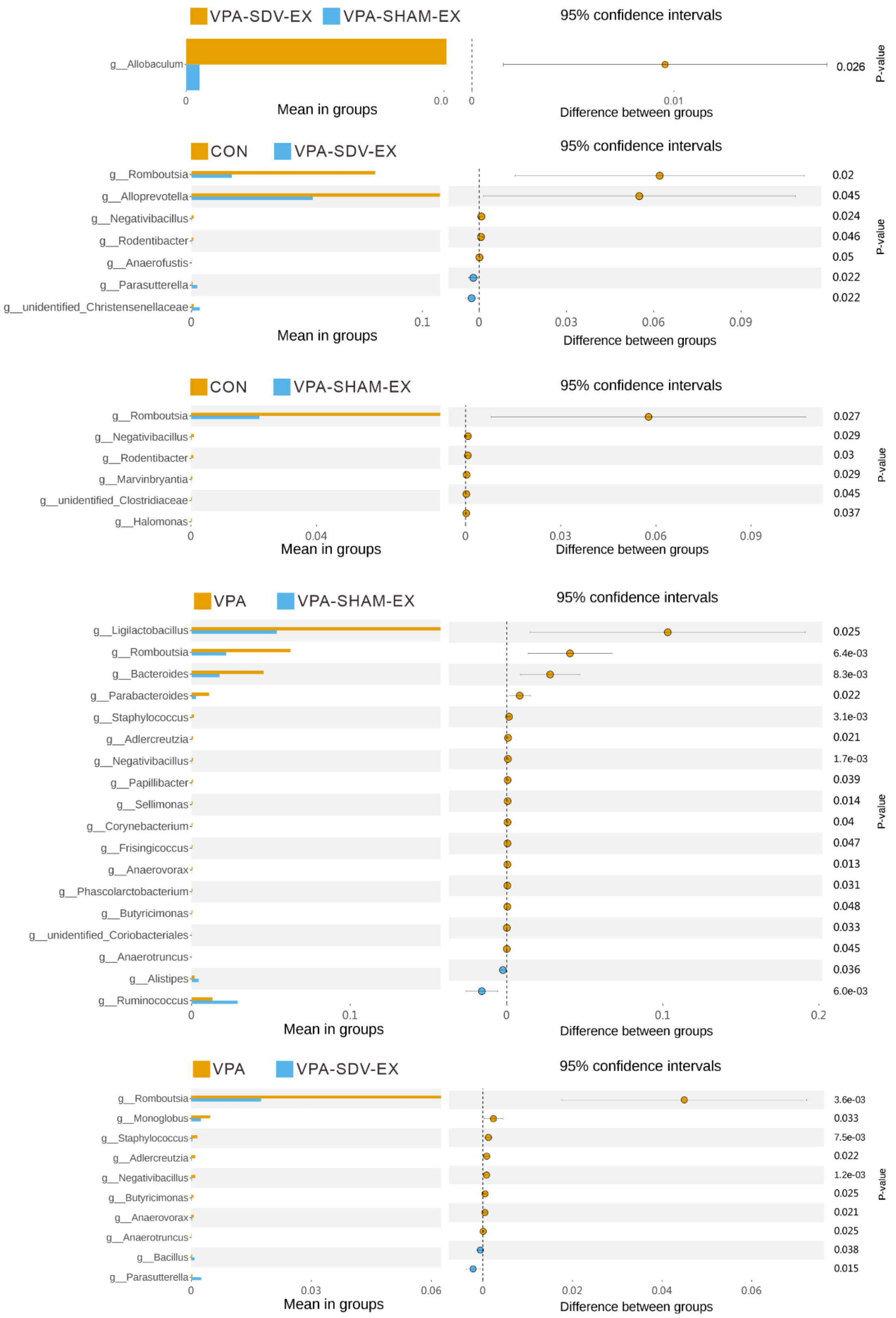

Supplementary Figure 10. Compositional differences at genus level in the gut microbiota across CON, VPA, VPA-VGX-EX and VPA-SHAM-EX groups.

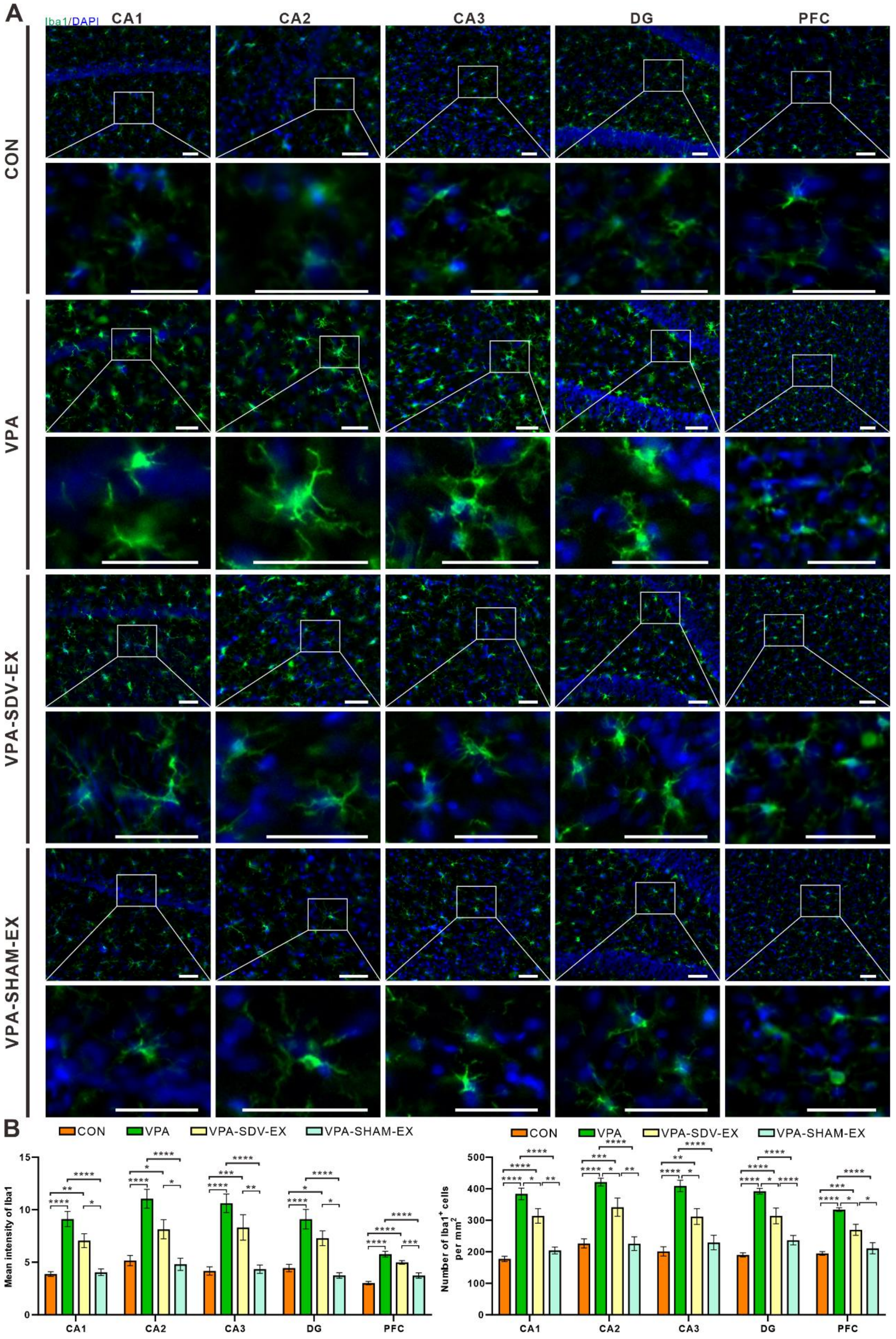

Supplementary Figure 11. Exercise following vagotomy affected microglia reactivity in the

hippocampus and prefrontal cortex of VPA-induced ASD-like rats. (**A**) Immunofluorescence for Iba1 (green) with DAPI (blue) nuclear counterstain in the CA1, CA2, CA3 subregions and dentate gyrus (DG) of the hippocampus as well as in the prefrontal cortex (PFC). (**B**) Mean intensity and number of $Iba1^+$ cells per mm^2. Data were expressed as mean ± SEM. Scale bar = 100μm, * $P < 0.05$, ** $P < 0.01$, *** $P < 0.001$ and **** $P < 0.0001$. All experiments were performed in triplicate, four animals from each group and twelve randomly selected images.

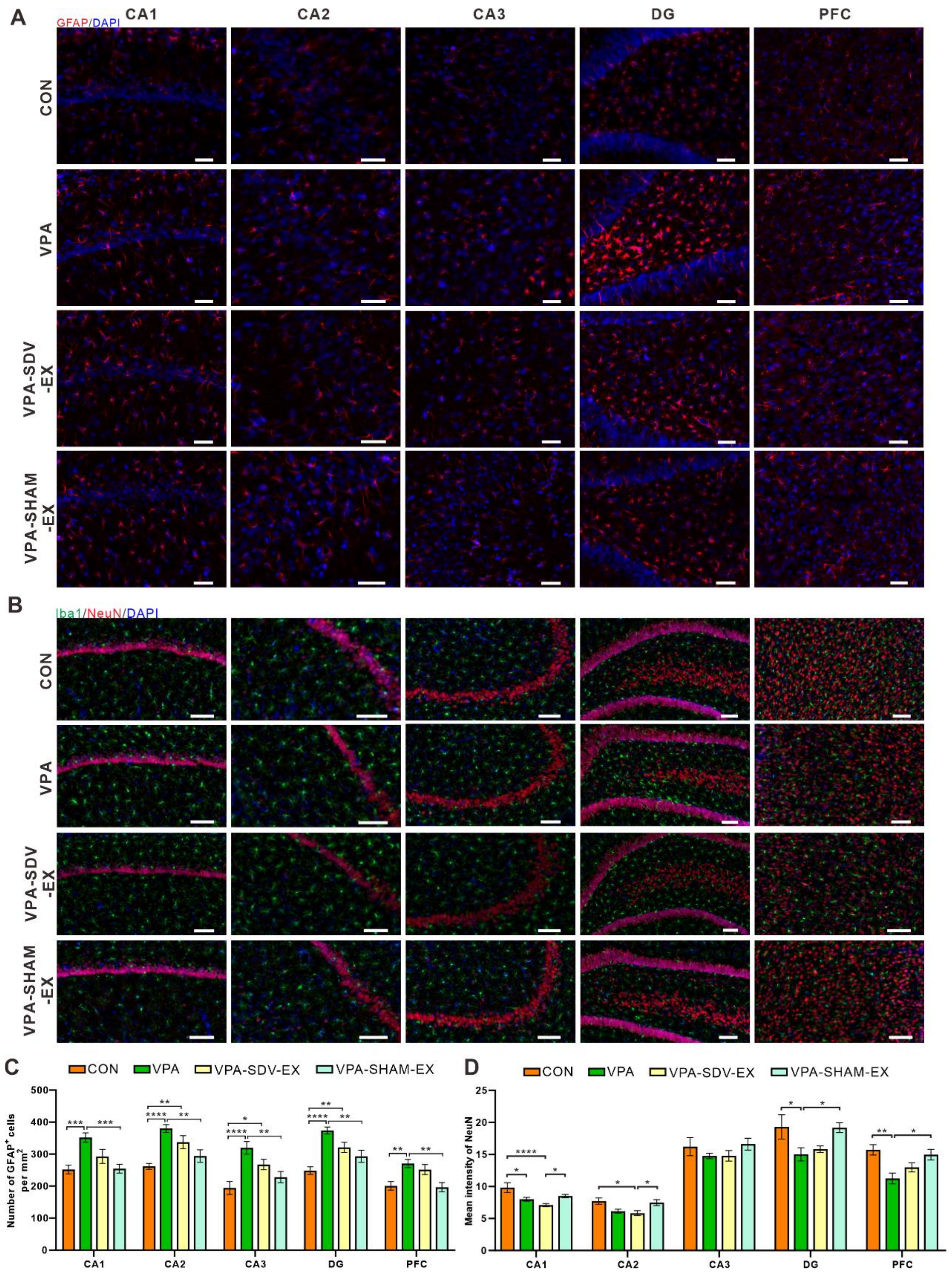

Supplementary Figure 12. Exercise following vagotomy affected astrocyte reactivity and neuronal expression in VPA-induced ASD-like rats. **(A)** Immunofluorescence for GFAP (red) with DAPI (blue) nuclear counterstain in the CA1, CA2, CA3 subregions and DG of the hippocampus as well as in the PFC. **(B)** Immunofluorescence for NeuN (red) with DAPI (blue) nuclear counterstain in

the CA1, CA2, CA3 subregions and DG of the hippocampus as well as in the PFC. **(C)** Number of GFAP$^+$ cells per mm^2. **(D)** Mean intensity of NeuN$^+$ cells per mm^2. Data were expressed as mean ± SEM. Scale bar = 100 μm, * *P* < 0.05, ** *P* < 0.01, *** *P* < 0.001 and **** *P* < 0.0001. All experiments were performed in triplicate, four animals from each group and twelve randomly selected images.
